# Unpaired Learning‐Enabled Nanotube Identification from AFM Images

**DOI:** 10.1002/advs.202512504

**Published:** 2025-12-25

**Authors:** Soyoung Na, Soobin Park, Younsu Jung, Jinhwa Park, Jimin Hong, Jihyun Lee, Albert Kim, Bongjun Kim, Gyoujin Cho, Eunju Cha, Seung Hyun Song

**Affiliations:** ^1^ Department of Electrical Engineering Sookmyung Women's University Seoul South Korea; ^2^ Institute of Quantum Biophysics Sungkyunkwan University Suwon South Korea; ^3^ Department of Medical Engineering University of South Florida Tampa FL USA; ^4^ Institute of Advanced Materials and Systems Sookmyung Women's University Seoul South Korea

**Keywords:** atomic force microscopy, generative adversarial network, nanotubes, unpaired training

## Abstract

Nanotubes, particularly single‐walled carbon nanotubes (SWCNTs), represent an important class of materials with valuable electrical, mechanical, and thermal properties. However, accurate characterization of nanotube network morphologies remains a significant challenge. We present a deep learning‐based approach for extracting nanotube morphologies from atomic force microscopy (AFM) images utilizing an image‐to‐image (I2I) translation framework based on cycleGAN with a specialized loss function that learns to transform AFM images containing nanotubes to images of pure substrates. By subtracting these translated substrate images from the original AFM images, we effectively isolated nanotube morphologies even on substrates with roughness exceeding the nanotube diameter. We validate our approach through physics‐based simulation studies and application to roll‐to‐roll printed carbon nanotube transistors on flexible polymeric substrates.Our method outperforms tranditional image processing and supervised learning models in sensitivity and accuracy for CNT network extraction. This improved characterization capability provides useful insights for optimizing the fabrication processes of CNT‐TFTs, supporting their development for flexible electronic applications. The methodology extends beyond carbon nanotubes to other nanomaterial‐based electronic devices, with future work aimed at expanding the model's generalization and integrating with real‐time AFM imaging.

## Graphical abstract

Identifying nanotubes on rough substrates is notoriously challenging for conventional image analysis. This work presents an unpaired deep learning approach that automatically extracts nanotube networks from atomic force microscopy images, even on complex polymeric surfaces used in roll‐to‐roll printing. The method outperforms human experts in accuracy and consistency, opening new possibilities for automated characterization in flexible electronics and nanomaterial research.

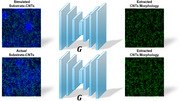

## 1 Introduction

Nanotubes represent a critically important class of nanomaterials that have revolutionized numerous fields including electronics, materials science, energy storage, biomedical applications, and environmental remediation. Their exceptional physical, chemical, mechanical and electronic properties, including high aspect ratios, excellent thermal and electrical conductivities, remarkable tensile strength, and tunable electronic band gaps, have established them as fundamental building blocks for next‐generation technologies [1, 2, 3, 4, 5]. Beyond carbon nanotubes, various inorganic nanotubes such as those fabricated from boron nitride, tungsten disulfide, titanium dioxide, and other transition metal dichalcogenides have demonstrated unique properties and applications ranging from superlubrication to catalysis and energy conversion systems [6, 7, 8].

Among various nanotube types, semiconducting single‐walled carbon nanotubes (SWCNTs) represent a particularly promising material for flexible and stretchable thin‐film transistors (TFTs). Previous works have demonstrated the viability of SWCNT‐based TFTs [9, 10, 11] and even wafer‐scale fabrication of modern microprocessors built from CNT‐based CMOS transistors [12], establishing them as an economical and flexible alternative to rigid silicon‐based technologies. The flexible and stretchable nature of nanotubes, particularly CNTs, offers significant advantages in fabricating flexible or stretchable electronics for various applications including wearable devices, biomedical implants, and flexible displays [9, 13, 14, 15, 16, 17].

However, despite these advancements, the fabrication of highly integrated nanotube‐based circuits faces challenges related to device‐to‐device variation. To achieve higher integration levels in nanotube‐based electronics, optimization of fabrication processes is essential. For nanotube‐based devices, performance is closely related to the densities and morphology of the nanotube network [18, 19]. Therefore, a thorough understanding of how different fabrication parameters affect network morphology is critical for device optimization. Atomic force microscopy (AFM) stands out as a valuable tool for characterizing nanotube morphologies, providing direct height information essential for understanding nanotube configurations and distributions. However, quantitative analysis of AFM images for extracting nanotube morphologies pose considerable challenges, particularly when the substrate roughness exceeds the nanotube diameter—a common occurrence in flexible and stretchable electronics. In such cases, conventional image processing techniques face significant limitations: [20].

In response to the limitations of manual analysis and inadequacies of conventional image processing techniques, we propose a deep learning‐based approach to extract nanotube morphologies from AFM im‐ages. Specifically, we employ an image‐to‐image (I2I) translation framework [21, 22], which is widely used when mapping between two different domains is required. Our approach focuses on learning the characteristics of the substrates rather than the nanotubes themselves, as substrate properties tend to be more homogeneous and easier to model effectively.

**FIGURE 1:**
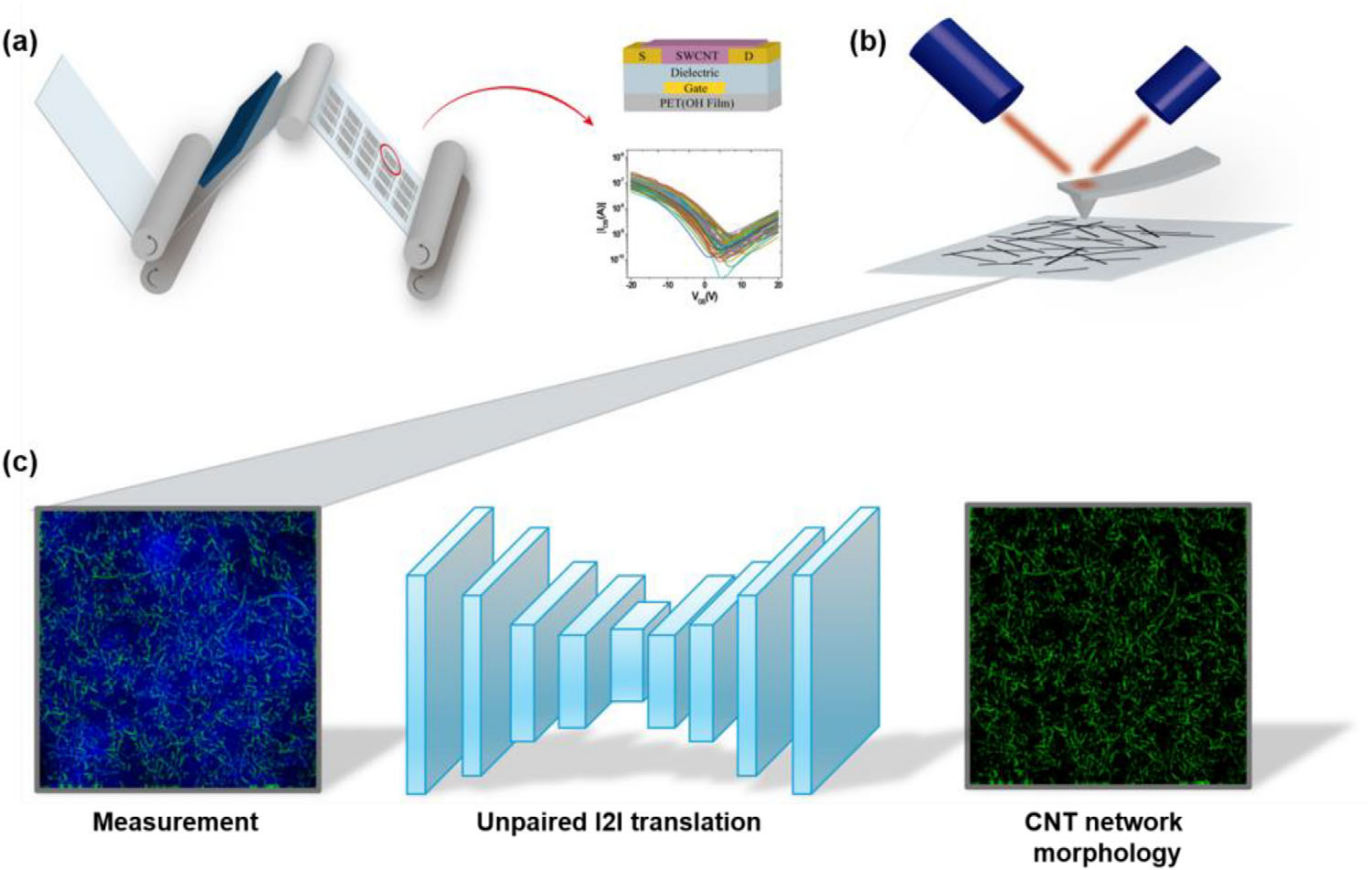
(a) R2R gravure printing process and electrical performance of CNT‐TFT. (b) Measurement of CNT network and substrate morphology using AFM (c) Extraction of CNT network morphology using the proposed unpaired I2I translation method.

We set two domains X and Y, where each set refers to the AFM images of substrates with nanotubes and the AFM images of pure substrates, respectively. We develop a framework based on cycleGAN [21] so that our model G learns the transformation from X to Y. By training the model on diverse substrate roughness patterns in the domain Y, the mapping can be estimated independently of substrate influence. With the trained model G, the morphology of the nanotube network can be extracted by subtracting the translated substrate image from the corresponding image containing nanotubes (Figure 1). In this work, we demonstrate the effectiveness of our approach using carbon nanotubes on rough dielectric substrates as a case study. This particular application showcases how our methodology can address a challenging scenario in nanotube‐based flexible electronics fabrication. The proposed approach not only promises enhanced accuracy in nanotube identification but also effectively addresses the challenges posed by substrate roughness, with potential applications to other types of nanotubes beyond carbon nanotubes. This work extends our previous study presented at European Signal Processing Conference (EUSIPCO) [23], which focused primarily on a deep learning‐based CNT extraction algorithm and included proof‐of‐concept validation using simulation datasets only. The present manuscript provides a complete and refined version of the previous study [23] by introducing an empirical validation framework that addresses the gap between simulation and real‐world applications. We further extend the evaluation methodology to incorporate real‐world datasets and benchmark the results against expert‐based reference standards. Extensive experimental results show that the proposed algorithm consistently improves performance and exhibits good potential for practical use.

## 2 Results and Discussion

### 2.1 Topographic Characteristics of R2R Printed CNT‐TFTs

For our study, we chose CNT‐TFTs on flexible polymeric substrate printed via roll‐to‐roll (R2R) process described in the previous work [24] as a platform for the development of deep learning‐based image processing tool for CNT network morphology characterization, since CNTs are deposited on a surface with high roughness and various morphological features. To briefly summarize the R2R fabrication process of CNT‐transistors, the gate electrode (Ag nanoparticles‐based ink) and the BiTiO3‐base dielectric layer are sequentially printed on polyethylene terephthalate (PET) substrate. The gate electrode and dielectric layers are dried in a dry oven at 150°C for 1 min. After drying, the active layer, comprising of SWCNTs, are printed atop of the dielectric layer. SWCNT ink was formulated based on the re‐ported method [24]. Finally, the source /drain electrodes were printed using an Ag nanoparticle‐based ink. Since SWCNTs exhibit p‐type characteristics in the ambient condition, additional printing is necessary to fabricate n‐type TFTs. For converting p‐type to n‐type, n‐doping ink poly(ethylene imine) with TiO2 nano‐power mixture in diethylene glycol solvent was printed on the SWCNT layer.

**FIGURE 2:**
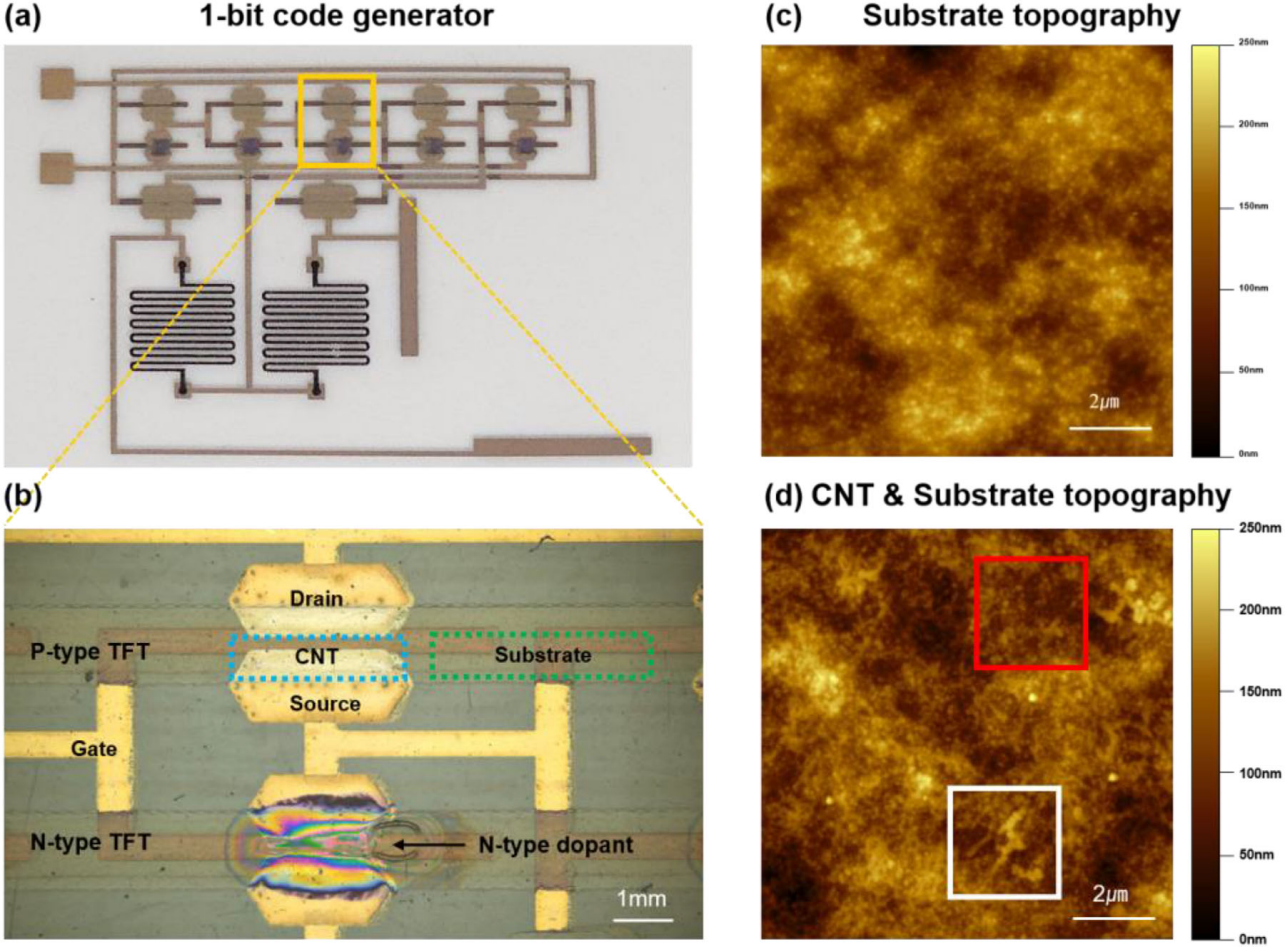
(a) R2R‐printed 1‐bit processor (b) Features of P‐type and N‐type transistor. (c) Substrate AFM topography and (d) CNT & Substrate topography. The white box indicates where the CNT network exhibits distinctly recognizable features and the red box indicates where the CNTs and the substrate are indistinguishable.

The photographic images of a 1‐bit code generator based on R2R‐printed SWCNT transistors and a representative transistors are shown in Figure 2a, b, respectively. The representative AFM topography images of the dielectric surface without CNT (green dashed box, Figure 2b) and the channel region (blue dashed box, Figure 2b), where CNT and dielectric layer are both present, and are shown in Figure 2c, d. The substrate topography image (Figure 2c) shows a highly uneven surface with craters and hills formed by the BTO nanoparticles and the rough surface of the PET substrate. For the topography image of the CNT deposited on the BTO dielectric layers, the large roughness of the underlying surface obscures the CNT network morphology, hindering the quantitative analysis of the morphology characterizations. Upon closer examination, wire‐like features are more clearly observed in some regions (white box, Figure 2d) compared to the other regions (red box, Figure 2d). Due to these complicating factors, we found that conventional junction identification methods based on traditional image processing techniques reported in the prior literature [20, 25] are insufficient due to the large surface roughness for intensity‐based (i.e. height of the topography image) classification methods (see Section S1).

In addition to the surface roughness, the CNT network morphology can be highly heterogeneous depending on the presences of CNT aggregates and contaminants, and the effect of CNT densities. To investigate the evolution of the CNT network morphology under varying densities of CNT, we carried out the fabrication of CNT‐TFTs under various conditions by modifying the SWCNT loading concentration in the ink ranging from 4 to 8 mg in 30 g butyl carbitol. For example, with the increasing CNT densities, it becomes more difficult to identify individual CNT strands as the aggregation of CNT strands are promoted (Figure S2a–d). On the other hand, AFM topography images with only the di‐electric substrates are relatively homogeneous (see Figure S2e–h) compared to the CNT topography images. In addition, the effect of the increasing CNT densities and corresponding evolution of the network morphology on transistor performances were clearly observed (Figure S3).

Based on this observation, we hypothesized that the CNT morphology features can be more effectively extracted by subtracting substrate features from the CNT topography images.

### 2.2 Proposed Training for CNT Extraction

**FIGURE 3:**
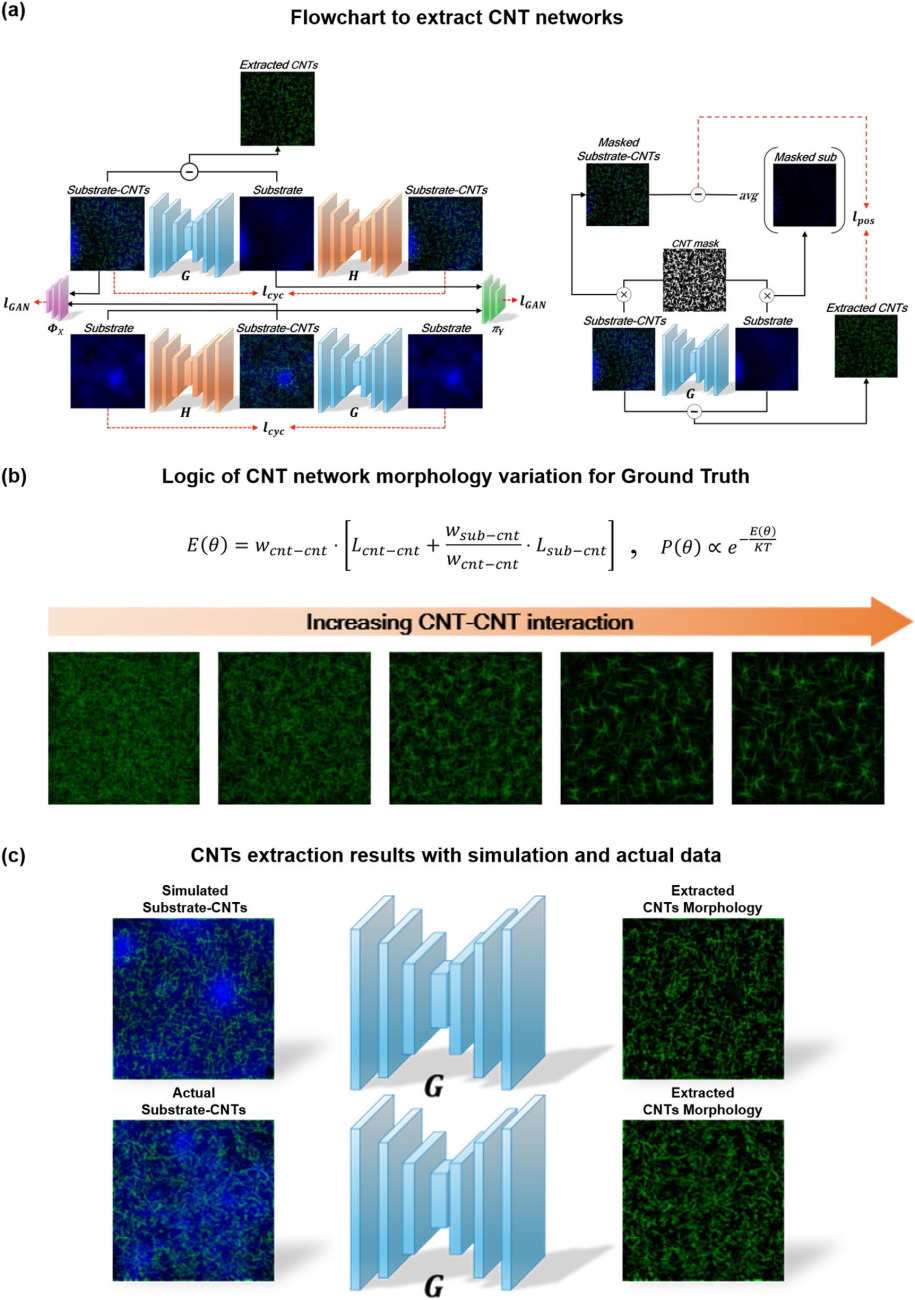
(a) Flowchart to extract CNT networks. (b) Logic of CNT network morphology variation for ground truth. (c) CNTs extraction results with simulation and actual data.

In order to accurately extract CNT morphology from AFM topography images in the presence of substrate roughness and varying topographic features, we propose a deep learning‐based algorithm based on I2I translation approach in Fig. 3(a). Since it is impractical and demanding to gather a paired dataset of AFM images of a substrate with and without CNTs for supervised learning, we exploit the unpaired training approaches which enables learning the mapping between two domains without the paired datasets.

With substrate with CNTs image domain X and substrate image domain Y, our model is trained to learn the mapping X↦Y by employing the cycleGAN framework [21]. Specifically, our model has two generators G:X↦Y,H:Y↦X and two discriminators $\phi_{X}$, $\pi_{Y}$. The discriminator aims to determine whether an image is translated by the model or from the target domain. On the other hand, the generator is trained to deceive the discriminator so that the discriminator can regard the output of the model as an image from the target domain. This scheme encourages generators to translate the input image indistinguishable from the target domain.

The overall optimization problem of the proposed method is given by:

(1) $$\begin{array}{c} \min_{G,H}\max_{\phi_{X},\pi_{Y}}\ell \left(G,H,\phi_{X},\pi_{Y}\right). \end{array}$$

Here,

(2) $$\begin{array}{ccc} l\left(G,H,\phi_{X},\pi_{Y}\right) & = & \ell_{\mathrm{GAN}}\left(G,\pi_{Y}\right)+\ell_{\mathrm{GAN}}\left(H,\phi_{X}\right)+\alpha \ell_{\mathrm{cycle}}\left(G,H\right)\\ & & +\beta \ell_{\mathrm{iden}\mathrm{tity}}\left(G,H\right)+\gamma \ell_{\mathrm{pos}}\left(G\right), \end{array}$$

where α,β and γ are hyper‐parameters. The adversarial loss $\ell_{GAN}$ is employed to make the generators, G and H, to produce results analogous to the images from the domain Y and X, respectively. We adopt least‐squares GAN (LS‐GAN) [26] loss as $\ell_{GAN}$ to train our model. The cycle consistency loss $\ell_{cycle}$ is used to push H(G(x))≈x and G(H(y))≈y. The identity loss $\ell_{identity}$ aims to force the output of the generator to be similar to the input image when the actual sample of the target domain is used as the input, i.e. G(y)≈y and H(x)≈x. Thus, an identity loss $\ell_{idnetity}$ was used to make the proposed model robust to the roughness of the substrate. It is worth commenting that we propose the novel loss function

$\ell_{pos}$ to encourage the generator G to selectively remove the CNT networks from images of substrate with CNTs on (Figure ):

(3) $$\begin{array}{c} \ell_{\mathrm{pos}}=E_{x∼p_{X}\left(x\right)}∥x_{\mathrm{cnt}}-\left(x\times M_{\mathrm{cnt}}-\mathrm{avg}\left[G\left(x\right)\times M_{\mathrm{cnt}}\right]\right)\;{∥}_{1}, \end{array}$$

where $M_{\mathrm{cnt}}$ denotes a binary mask with the value 1 for pixels with CNTs and 0 otherwise. avg[*·*] is an operator that averages the values of all pixels to estimate the heights of the substrate. Therefore, the minimization of $\ell_{pos}$ enforces the CNT network image $x_{\mathrm{cnt}}$ to provide the CNTs’ height and not the substrate's height only for the regions where the CNTs are actually present. Therefore, with the trained model G∗ via (2), the CNT networks can be extracted by subtracting the translated substrate image from the image of substrate with CNTs on:

(4) $$\begin{array}{c} x_{\mathrm{cnt}}=x-G^{*}\left(x\right). \end{array}$$

This leads to the efficient extraction of CNT networks in a much shorter time than with conventional algorithms, without the need for experts.

### 2.3 Ground‐Truth Generation via CNT Network Simulation

One of the key challenges in developing the deep learning model for the CNT morphology extraction application is the lack of robust ground‐truth to validate the accuracy of the extracted CNT features. To address the issue, we performed CNT network simulation based on the physical and experimental insights regarding the CNT deposition on the dielectric substrate to obtain ground‐truth images that closely resemble the actual AFM images of the CNT‐TFTs. Firstly, we examined the evolution of the CNT network morphology as a function of CNT density and the surface energy. Since the deposition of CNTs in R2R process is carried out as a form of ink where CNTs are dispersed in a carrier solvent, the resulting network morphology should, theoretically, be heavily influenced by the interaction energies between the substrate and CNT (*w_sub−cnt_*) and between CNTs (*w_cnt−cnt_*) [27, 28]. We have also experimentally confirmed the influence of the substrate properties by inkjet printing SWCNTs with varying densities on three different substrates; 3‐Aminopropyltriethoxysilane (APTES) treated, oxygen plasma treated, and untreated silicon oxide surface. In summary, the CNTs tend to spread without aggregating on the APTES‐treated substrate, whereas CNTs deposited on the untreated substrate tend to aggregate. In addition, many CNTs were aligned in the same angular orientation when forming aggregates, likely to maximize the interaction lengths between CNTs to lower the overall energy (Figures S4 and S5).

Based on the prior literature [27, 28] and our experimental observations, we established a simplified model for CNT deposition. Deposition occurs when a single strand of CNT comes into a contact with the substrate; typically, due to the high mobility of CNT in the solvent, the deposition occurs following the equilibrium determined by the competition between thermal motions and the overall energy of the system [28], given as equation below, where the increase in the overall energy by the deposition of a single strand of CNT is given by the interaction lengths and interaction energies. Thus, we assume that the CNT, once it comes in contact with the substrate can no longer be displaced but is free to rotate; depending on the orientation of the CNT, the energy of system is determined by the length of interaction and the energy of interaction.

(5) $$\begin{array}{c} E\left(\theta \right)=w_{\mathrm{cnt}-\mathrm{cnt}}\cdot \left[L_{\mathrm{cnt}-\mathrm{cnt}}+\frac{w_{\mathrm{sub}-\mathrm{cnt}}}{w_{\mathrm{cnt}-\mathrm{cnt}}}\cdot L_{\mathrm{sub}-\mathrm{cnt}}\right] \end{array}$$

Utilizing these properties, we calculate the relative probabilities associated with the angles of a single strand of CNT based on the Boltzmann probability determined by the configuration energy E(θ) as a function of *w_sub−cnt_* and *w_cnt−cnt_* (Figure 3(b)). The simulations of the CNT network topography are performed by repeating the following deposition process. Firstly, the coordinate of a SWCNT is randomly generated and all the energies and associated probabilities are calculated for all angles. Then the deposition angle is randomly selected (note that not all angles are equally probable).

(6) $$\begin{array}{c} P\left(\theta \right)\propto \exp-\frac{E\left(\theta \right)}{K_{B}T} \end{array}$$

We firstly validated our simulation model by reproducing the experimental observations of network characteristics of CNTs deposited on three different substrates with different CNT densities (Figure S6). Taking the model, we varied the length, quantity, and energy of CNTs through simulation method to generate CNTs network images. Combining these with actual substrate AFM images allowed obtaining ground‐truth images closely resembling the actual CNT AFM image (Figure S7).

Following the validation of the simulation process, we carried out the data preparation for the model training by firstly setting the parameter boundaries. In order to account for various inhomogeneity arising from the fabrication processes (i.e. different CNT densities, presence of pre‐exisitng aggregations, etc), we set a large range of parameters for the data generation as listed in Table 1 to ensure that the generated images resemble the experimental data closely. During the data generation step, the values within the simulation range is randomly selected.

**TABLE 1:** Data set for generation simulation image.

$\frac{w_{sub-cnt}}{w_{cnt-cnt}}$	Number of Line	Number of Arc	Number of Sector
5–20	500–3000	166–1000	334–2000

Line Length	Arc length	Curvature	angle
40–60	0–10	0–360	30–60

### 2.4 CNT extraction using simulation data

To evaluate the performance of our model on the simulation data, we generated 50 images of CNT net‐works under the conditions described in the preceding section and added them to the 20 substrate im‐ages with various textures to construct the test dataset of 1000 images.

Using the dataset, we compared our model against the Otsu‐based method [20], the supervised learning‐based method, and cycleGAN [21] to evaluate the performance of our model. For the Otsu‐based method [20], the low‐pass filter was applied to the raw AFM image to remove the fast scan artifacts. Then, the local Otsu algorithm was used to create a binary mask based on the presence of CNTs. The CNT net‐works can be extracted by multiplying the binary mask with the corresponding substrate‐CNT image.

Since a paired data set containing images of substrates with CNTs and the corresponding CNT networks is available for the simulated dataset, we also carried out supervised learning. In particular, the network was trained to capture the CNT networks directly from the given substrate‐CNT images. To verify the feasibility of the proposed loss function $\ell_{pos}$ in (3), the original cycleGAN [21] model was also used as a comparison study. Note that the cycleGAN model for comparison is identical to the proposed algorithm in terms of network architecture and training details, except for the use of $\ell_{pos}$ in the objective function (refer to cycleGAN Section S6 for more details).

**FIGURE 4:**
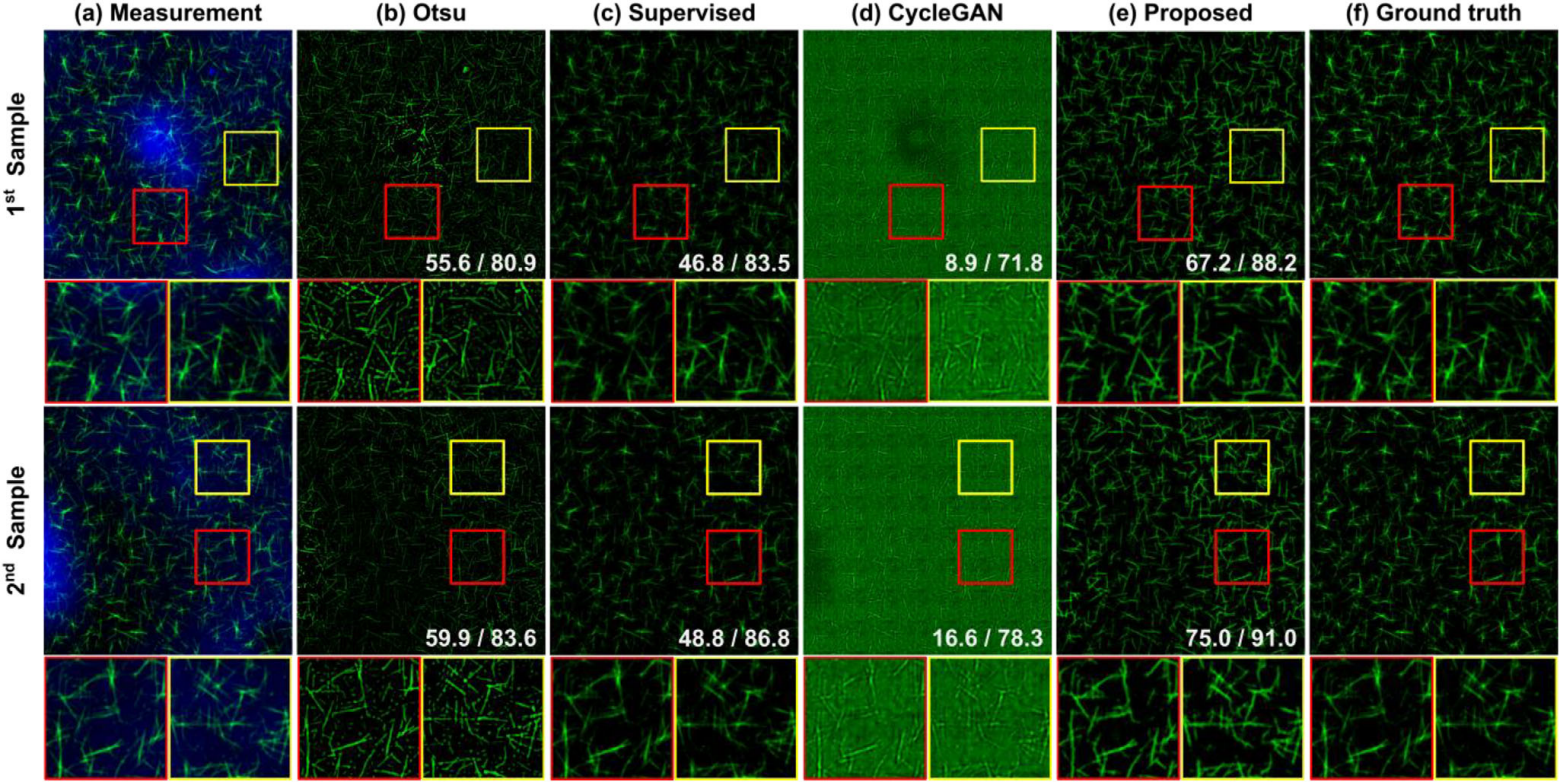
CNT extraction results using simulation dataset: (a) Substrate‐CNT image, (b) Otsu‐based method [20], (c) supervised learning method, (d) cycleGAN [21], (e) proposed method, and (f) ground‐truth CNT networks. The values in the corners are sensitivity / accuracy for each image with respect to the ground‐truth data.

Figure 4 illustrates a qualitative comparison between our model and other algorithms. Figure 4a shows representative test dataset generated by adding a real substrate topography image (colored in blue) and simulated CNT network (green). As depicted in Figure 4b, numerous dot‐like features can be observed from the extracted CNTs (inside both red and yellow boxes), which are the BTO‐nanoparticle aggregates from the substrate. Since the Otsu‐based algorithm identifies CNTs based on intensity differences, it often results in incorrect identification of CNTs. Therefore, the substrate regions with high intensities can be classified as CNTs even when no CNTs are present. In particular, these characteristics can be found in the red box of the 1st sample in Figure 4b. Furthermore, since the conventional Otsu‐based algorithm extracts CNT networks without predicting the substrate height, the results include both the substrate and the CNT network, leading to a higher intensity compared to other algorithms. In Figure 4c, the results using the model trained in a supervised learning manner are presented. With this model, more accurate shape and height of the CNT networks can be provided compared to the Otsu‐based algorithms. However, the supervised learning‐based model exhibits significantly degraded performance on real experimental data, which is due to its limited generalization capability stemming from the difference between the simulated and actual images. This issue is discussed further in section 2.6, where we verify the algorithms in analyzing the actual data.

To mitigate this issue, we adopt the cycleGAN [21] framework, which enables to train the model without the paired dataset. Figure 4d represents the results of extracted CNT networks using cycleGAN [21].

Even though the network architecture of cycleGAN was the same as that of the proposed algorithm, the trained model suffers from the prediction of the substrate and the extraction of the CNT networks.

Specifically, only the regions consisting of clusters were classified as the substrate, and all remaining regions were incorrectly classified as CNTs. Note that the sensitivity of the results with cycleGAN of the 1st sample is 8.9 %, which is significantly lower than that of Otsu‐based algorithms. As shown in Figure 2c, d, the high texture similarity between the substrate images with CNTs and the pure substrate images makes it difficult for the model to learn distinguishable features between the two domains. These results confirm that the conventional cycleGAN needs to be further modified to enable the precise extraction of CNT networks.

The CNT networks extracted using the proposed method are presented in Figure 4e. The proposed algorithm outperforms competing methods, especially regarding the continuity of the CNTs and residues of the particles on the substrates. As can be seen in the zoomed regions in Figure 4e, the proposed method provides a detailed structure of the CNT networks that cannot be investigated in Figure 4b, d. Since the ultimate goal of the algorithms is to investigate the morphology of CNT networks, such factors subsequently impact the analysis. The more precise estimation of the CNTs also results in higher accuracy and sensitivity than other algorithms, even compared to the supervised learning‐based algorithm. By comparing the results in Figure 4d, e, it is noteworthy that the pro‐posed loss function $\ell_{pos}$ significantly contributes to the model's effectiveness in identifying the CNT net‐works from images of the substrate with CNTs even with the unpaired dataset.

For quantitative evaluation, pixel‐based classification metrics were utilized. Specifically, we compared the binarization mask generated from the extracted CNT networks $x_{\mathrm{cnt}}$ based on the presence of CNTs with the ground‐truth CNT network mask $M_{\mathrm{cnt}}$. We computed accuracy, sensitivity, specificity, and precision pixel‐wise. The metrics are acquired based on true positive (TP), true negative (TN), false positive (FP), and false negative (FN) defined in Table 2. The metrics are defined as follows:

(7) $$\mathrm{Accuracy}=\frac{TP+TN}{TP+FP+TN+FN}$$

(8) $$\mathrm{Sensitivity}=\frac{TP}{TP+FN}$$

(9) $$\mathrm{Specificity}=\frac{TN}{TN+FP}$$

(10) $$\mathrm{Precision}=\frac{TP}{TP+FP}$$

**TABLE 2:** Definition of the abbreviations.

	Actual CNTs	Actual No CNTs
Predicted CNTs	True Positive (TP)	False Positive (FP)
Predicted No CNTs	False Negative (FN)	True Negative (TN)

Table 3 shows the quantitative comparisons with competing algorithms. Our method outperformed other methods by 1.1–12.0% and 23.0–65.6% in terms of accuracy and sensitivity, respectively. The com‐parison between the results with cycleGAN and the proposed method once again verifies the important role of the proposed loss function $\ell_{pos}$. The proposed loss function enforces the network to estimate the height of the substrate accurately even when CNTs are present, which enables a more exact prediction of the morphology of the CNT networks. One note is that higher accuracy and sensitivity can provide more information about existing CNTs and facilitate detailed analysis of CNT networks. Therefore, the experimental results showed that the proposed method not only offers better performance in extracting CNT networks but also is more sensitive to the presence of CNTs on the substrate.

**TABLE 3:** Quantitative comparison using simulation dataset. The results are mean values of 1000 test results.

	Accuracy [%]	Sensitivity [%]	Specificity [%]	Precision [%]
Otsu [20]	82.3	47.4	94.5	78.7
Supervised	88.2	54.2	**99.6**	**97.7**
cycleGAN [29]	77.3	11.6	99.1	86.0
Proposed	**89.3**	**77.2**	93.7	80.5

In contrast, the proposed algorithm provided relatively lower performance regarding specificity and precision compared to the other algorithms. As defined in (9) and (10), specificity and precision are associated with the value of FP, i.e., the number of pixels at which the model falsely indicates the presence of CNTs. Consistent with the qualitative results demonstrated in Figure 4, the supervised learning‐based method and cycleGAN extract fewer CNTs than shown in the ground‐truth. Therefore, the FP of the supervised learning‐based model and cycleGAN are close to 0, resulting in high values for specificity and precision. In addition, since the proposed method provides more continuous CNT networks, it tends to predict the tails of the CNT networks as long with a relatively low height. However, when constructing a binarization mask based on the presence of CNTs to calculate sensitivity and precision, the predicted height of the CNTs is not considered at all, which leads to a lower value in terms of two metrics com‐pared to the supervised learning‐based method and cycleGAN. As shown in Figure S11, the threshold value for generating the binary mask is one of the major hyperparameters to control the trade‐off be‐tween specificity and sensitivity. In particular, lowering the threshold increases sensitivity, while raising it increases specificity. More fundamentally, this issue can be addressed by developing an additional regularization that calculates the reliability of the presence of CNTs based on the height value of the extracted CNTs during training, which is being considered as future work.

Figure S10a shows the box plots for the accuracy, sensitivity, specificity, and precision of the CNT net‐work analysis carried out by the proposed model and supervised learning‐based model. We observed that the evaluations on the simulated data exhibit a high prevalence of outliers. In particular, we found that the outliers were mainly because of the misidentification of the substrate features as CNT, which was attributed to the presence of contamination on the surface. Figure S10b illustrates the comparison between the image showing good accuracy (the 1st sample) and the degraded substrate image (the 2nd sample), which were utilized to create the simulation data for evaluation. We note that both substrate images were taken from a single‐scan image. To identify the cause of the performance degradation, we examined the AFM phase images, which serve as a tool for analyzing the characteristics of substrate materials, particularly through the interaction between the tip and the sample. The AFM phase distribution of the image shown in the first sample of Figure S10b, where we observed performance degradation, was wider compared to that of the second sample of Figure S10b, where the algorithm showed good performance. In addition, the phase distribution showed the presence of a double peak. Since the pure substrate should only be composed of BTO‐nanoparticles, the phase distribution is expected to follow a narrow Gaussian. Thus, in turn, the fact that the phase distribution within the red box can be better fitted using double Gaussian fitting strongly suggests the presence of contaminants other than the substrate itself. In contrast, the second sample can only be fitted using a single Gaussian fitting, indicating a more homogeneous surface without the presence of contaminants. Thus, we tentatively assign the contaminants as the cause of degradation of the performance of the proposed model, which is also a current limitation. Our proposed nanotube identification method performs well on substrates with minimal contaminants. However, polymeric contaminants frequently introduced during fabrication and solvent processing can degrade performance by spreading and aggregating to form additional topographical features [30]. While our method can distinguish certain contaminants, this capability remains limited. Although proper cleaning processes help mitigate contamination [31], residual contaminants often persist.

Because our method detects non‐substrate features, it gains versatility across diverse conditions but be‐comes susceptible to contamination artifacts. To address this limitation, we propose utilizing additional AFM data, such as friction and phase contrast information, which would provide the algorithm with bet‐ter discriminative power between genuine nanotube features and contaminants. We aim to explore this approach in future work.

### 2.5 Performance Comparison of Proposed Algorithm against Expert and Student CNT Junction Identification

**FIGURE 5:**
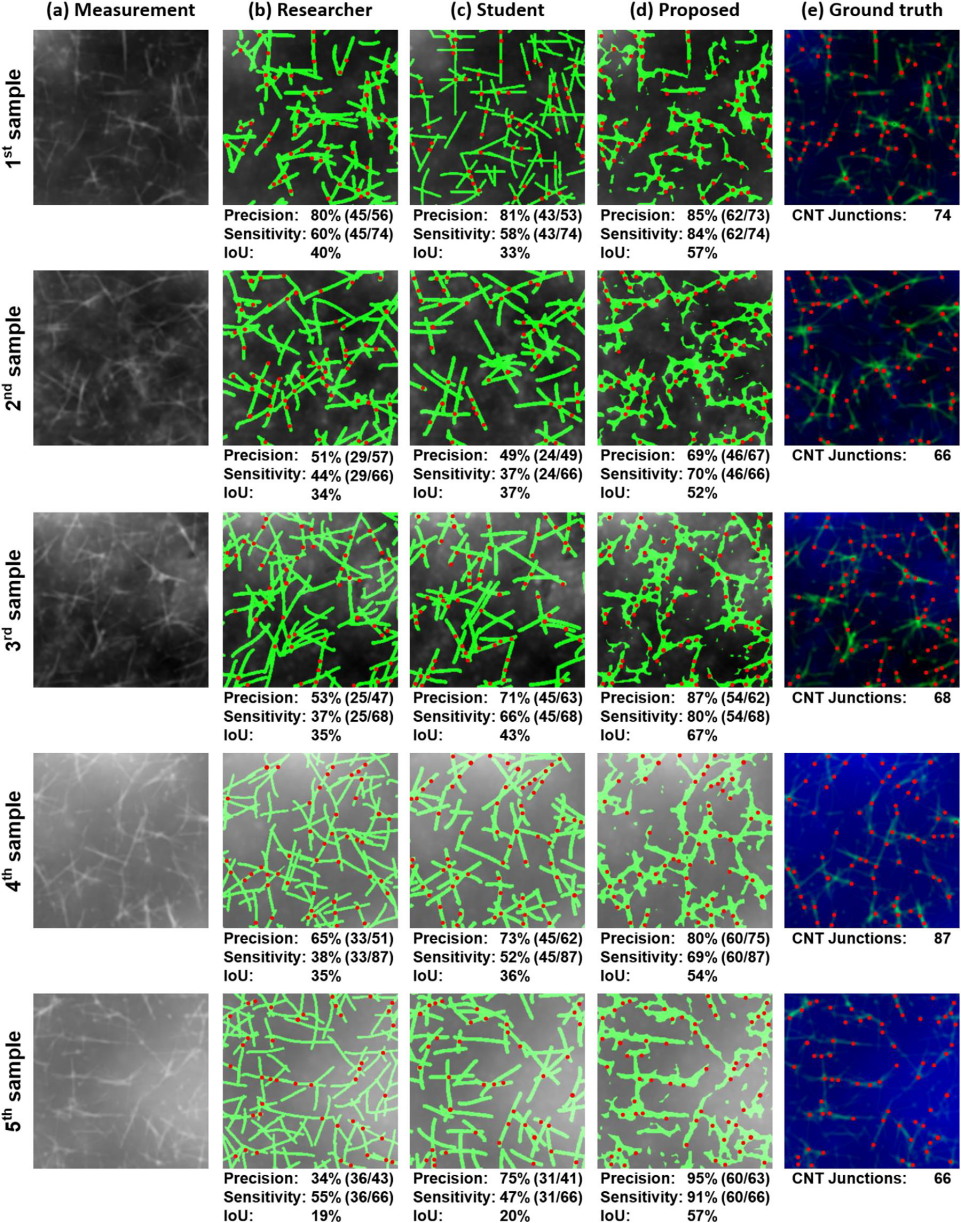
Comparative analysis of CNT junction detection across five different samples. (a) Measurements with different surface roughness. (e) Ground‐truth annotations showing actual CNT junctions. (b) Results from an expert researcher show moderate sensitivity (37–60%) but a wide variance in precision (34–80%) and lower IoU (19–40%). (c) Results from a student operator show variable performance with precision of 49–81%, sensitivity of 37–66%, and IoU of 20–43%. (d) Results from our proposed algorithm demonstrate high precision (69–95%), sensitivity (69–91%) and IoU (52–67). The proposed method consistently outperforms manual detection by both experienced and novice operators when identifying CNT junctions on rough flexible substrates.

Following the validation of the CNT extraction using the proposed algorithm, we evaluated its performance against manual analysis by human operators with different levels of expertise. Figure 5 presents a comprehensive comparison of CNT extraction by the proposed algorithm, an expert researcher, and a student, respectively, on five representative samples generated by superimposing simulated data on the topography of the actual substrate. For a fair comparison, both our model and the human annotators identified solely CNTs on the simulated data of substrates containing CNTs and the CNT junctions. The resulting masks subsequently were multiplied by the ground‐truth CNT topography to isolate the identified CNT regions. Since the simulated CNT data were generated such that individual CNTs have a height of around 30 nm, regions with height values equal to or greater than 60 nm were considered CNT junctions. For enhanced visual clarity, Figure 5 highlights the CNT junctions, where intersecting CNTs form distinct angular deviations, indicated by circular markers.

The ground‐truth data were generated using the same procedure, in which the junctions are annotated from the simulation CNT topography. We compared the number of CNT junctions to validate the junction‐specific capability and to assess the network characterization performance in terms of precision, sensitivity, and the Intersection over Union (IoU), which quantifies the ratio of the overlapping area to the combined area of the ground‐truth and the model's prediction. We computed IoU in pixel‐wise manner to assess how accurately the predicted regions of CNT presence align with the annotated ground‐truth.

As shown in Figure 5a, for each sample, 66–74 actual CNT junctions are identified in the ground‐truth data, which serve as the reference standard for performance evaluation. Figure 5d) shows that the proposed algorithm consistently achieved superior precision, ranging from 69% to 95%, with an average of 83% across all samples. The sensitivity measurements, which range between 69% and 91%, demonstrate that the proposed method enables to identify the majority of the junctions actually present in the samples. These results confirm that our algorithm can estimate the precise height of CNTs, which is highly helpful in analyzing CNT morphology. Furthermore, the model yields satisfactory IoU values, particularly considering that it was computed at the pixel level. In contrast, even the expert operator exhibited inconsistent precision (34–70%) and low sensitivity (37–60%), as demonstrated in Figure 5b.

Figure 5c represents the results from the student operator, which provided moderate precision (49–81%) coupled with limited sensitivity (37–66%), highlighting the difficulty and subjectivity in manually classifying junctions. The tendencies of the individual operator are consistent in the IoU results. The expert operator generally recognizes more instances than the ground‐truth, leading to a larger union and consequently a lower IoU. Conversely, the student operator tends to detect fewer CNTs than the ground‐truth, resulting in a relatively moderate IoU value. These results imply that human analysis, even from experienced researchers, is prone to over‐detection and identifies multiple false positives, potentially leading to inaccurate network characterization. The superior performance of our proposed algorithm demonstrates that it can standardize CNT junction detection, which significantly reduces operator‐dependent variations and provides more reliable results for subsequent analysis of CNT network properties. In terms of processing efficiency, our model took only 0.4 seconds to extract CNTs from a 256×256 image, while the human annotators took an average of 15 min for the same task. Moreover, it is worth noting that the proposed model is robust to substrate roughness, as evidenced by consistently accurate results for all samples with different roughness levels in Figure 5. This highlights the valuable advantage of our automated approach in terms of both speed and scalability. These improvements are particularly beneficial for developing reproducible characterization methods necessary for advancing CNT‐based flexible electronic devices.

### 2.6 CNT Extraction Using Actual Data

**FIGURE 6:**
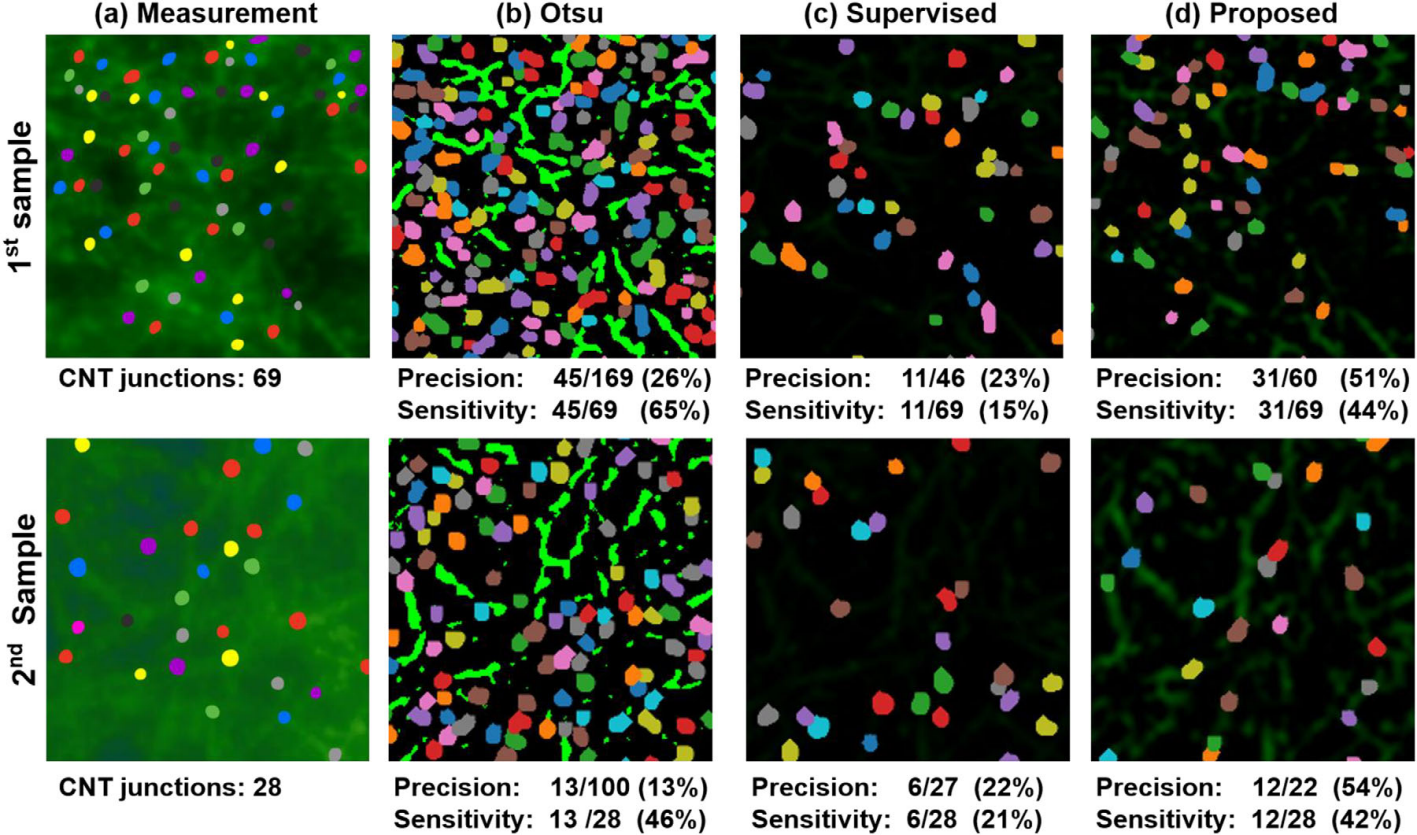
Extraction of CNT junctions using image processing on two actual samples: Analysis of precision and sensitivity for inference results obtained through (b) Otsu‐based method [20], (c) supervised model, and (d) proposed algorithms, based on manually extracted CNT junction counts with (a) the ground truth.

After performing a performance analysis based on the simulation data, we proceeded to evaluate the performance of the actual AFM image using the Otsu‐based method, the supervised learning‐based model, and the proposed algorithm. As demonstrated in Figure 5, it is very difficult to perfectly identify all CNT junctions in the images, even for experienced researchers. However, for real data, there is no way to recognize CNT junctions from the images without the help of experts. Thus, the CNT junctions were identified manually by experienced experts independently for the quantitative comparison. Figure 6 illustrates the precision and sensitivity results based on the number of CNT junctions extracted via thresholding, evaluated against the CNT extraction results of each algorithm. The CNT junctions from the results of the CNT extraction are marked following the procedure described in Section 2.4.

Figure 6b shows that the Otsu‐based method, compared to the proposed algorithm, exhibits higher sensitivity but lower precision. This indicates that while the Otsu‐based method detects more CNT junctions, it also tends to misidentify BTO substrate particles as CNTs, leading to an increased number of false positives. As shown in Figure 6c, the supervised learning‐based model exhibits notably inferior performance on actual data, in contrast to its results on simulated data. In particular, as depicted in the 1st sample in Figure 6c, the supervised learning‐based model predicts significantly fewer CNTs than other methods, leading to inaccurate junction estimation compared to the ground‐truth. Since supervised learning aims to learn a mapping between input data and corresponding labels, the model may be biased toward samples in the training dataset. This leads to a difficulty in covering the entire data distribution, which is particularly critical in our task as the data exhibit much more diversity compared to other natural image datasets due to the roughness of the substrates. Thus, we employed the cycleGAN framework, which enables data distribution matching between unpaired datasets rather than learning a direct mapping between paired data. In addition, to better capture the features of CNTs, we designed the novel loss function $\ell_{pos}$. As a result, the proposed algorithm successfully extracted only CNTs, achieving high precision and sensitivity, as shown in Figure 6d. The comparison indicates that the proposed algorithm is better at handling real‐world data compared to supervised learning, even if the data contains abnormalities and artifacts. Therefore, we confirm that the proposed algorithm enables the extraction of CNT junctions from actual AFM images with both high precision and sensitivity.

In addition, we argue that our method generalizes robustly across different AFM instruments and substrate types. While absolute height measurements vary between instruments and tips due to instrumental differences, the relative topographical features and morphological characteristics detected by our algorithm remain consistent. Regarding substrate generalization, nanotube identification is increasingly straightforward on atomically smooth substrates such as mica or low‐roughness surfaces like silicon oxide. The substrate used in this work—nanoparticles forming a dielectric layer on a plastic substrate—presents a particularly challenging case due to complex underlying topography. Success on this substrate suggests comparable or superior performance on less complex surfaces. Regarding CNT inks, we expect minimal sensitivity to ink variations, as our algorithm detects non‐substrate topographical features rather than specific nanotube characteristics. This allows generalization across different CNT sources, concentrations, and potentially other nanotubes types. However, performance may be affected by extreme conditions such as heavy contamination or severe aggregation, as discussed previously.

## 3 Conclusions

In this work, we developed and demonstrated the application of a deep learning‐based image‐to‐image translation approach for accurately extracting the carbon nanotube (CNT) networks from AFM topography images of R2R‐printed CNT‐FETs, overcoming the challenges posed by substrate roughness and varying topographic features. By leveraging the cycleGAN architecture with a novel loss function, the proposed method efficiently isolated CNTs from complex substrate‐CNT images, facilitating precise analysis of CNT morphologies. The developed methodology was validated through physics‐based simulation studies and application to real datasets, highlighting its robustness and accuracy compared to traditional image processing techniques and supervised learning models. The deep learning approach significantly improved the sensitivity and accuracy of CNT network extraction, providing a more detailed and reliable representation of the network morphology.

Our findings underscore the critical impact of CNT network morphology on the performance of flexible electronics and the necessity for advanced characterization techniques. The proposed deep learning framework offers a powerful tool for optimizing the fabrication processes of CNT‐TFTs, paving the way for their broader adoption in cost‐effective and flexible electronic applications, particularly in the burgeoning fields of IoT and smart packaging. Future work will focus on further enhancing the model's generalization capabilities to handle a wider variety of substrate conditions and extending the application of this method to other types of nanomaterial‐based electronic devices. Additionally, integrating this approach with real‐time AFM imaging could provide invaluable insights into the dynamic processes governing CNT network formation and evolution during fabrication.

## 4 Experimental Section

### 4.1 AFM Image Acquisition

The AFM images of the R2R‐printed CNT transistors were acquired using an AFM (XE‐7, Park Systems). We characterized five CNT‐TFTs printed using inks with varying CNT concentrations from 4 mg to 8 mg. Each image was captured at a resolution of 1024×1024 pixels, covering a 10 *µ*m × 10 *µ*m area in AFM tapping mode (PPP‐NCHR, Nanosense). We note that AFM measurements of nanotube height and width are subject to tip convolution effects and reflect the convoluted topography rather than the true nanotube dimensions. However, since our algorithm is trained and validated on experimental AFM data, this approach is appropriate for practical nanotube identification applications. The morphological features extracted by our method therefore represent the AFM‐measured characteristics that users will encounter in real experimental settings.

### 4.2 Data Preparation for Model Training

Acquiring over 1000 AFM images of CNT‐TFTs for model training is challenging. However, a few images cannot reflect the diverse characteristics of CNT network morphologies, resulting in performance degradation. Thus, we utilized AFM images captured in hand only for the substrate domain Y, while the data for the substrate with CNTs on domain X were generated via CNT network simulation. For simulation, we consider two factors, such as CNT concentration and interaction energies between substrate and CNT and between CNTs. For modeling CNTs networks with concentrations ranging 4mg to 8mg, we varied the CNT quantity, length, height and bending angles as illustrated in Table 1.

We generated 100 CNT network images through the simulation and individually added them to 33 substrate images to create 3300 images for the domain X. During model training, the information where the CNTs are present is needed to calculate the proposed loss function $\ell_{\mathrm{pos}}$ in (3). Therefore, the mask $M_{\mathrm{cnt}}$ was created through binarization in the aforementioned process. Specifically, the value of the mask $M_{\mathrm{cnt}}$ is one at the pixels where CNTs exist and zero otherwise. The substrate images are randomly augmented by a combination of flipping and rotation to create 3300 images for the domain Y. The dataset of each domain consists of 3300 images, each with 1024 *×* 1020 resolution.

Note that the differences in features between the two domains, X and Y, were very slight, as shown in Figure 2c, d. This issue leads to the model having difficulty distinguishing between the two domains. Thus, an additional pre‐processing step is required to maximize the discrepancies between the two domains. In particular, the substrate images are blurred using the Gaussian kernel with standard deviation of four and rescaled to ensure the original intensity of the data. With the processed substrate images, the model can learn the typical characteristics of smooth textures in substrate images and rough textures in substrate images with dispersed CNTs, facilitating the learning process.

### 4.3 Details of Network Training

Our objective function in (2) is similar to the loss function in [21], except for $\ell_{\mathrm{pos}}$. In particular, the loss functions $\ell_{\mathrm{GAN}}$ in (2) is given by:

(11) $$\begin{array}{ccc} \ell_{\mathrm{GAN}}\left(G,\pi_{Y}\right) & = & E_{y∼p_{Y}\left(y\right)}\left[\log\pi_{Y}\left(y\right)\right]\\ & & +E_{x∼p_{X}\left(x\right)}\left[\log\left(1-\pi_{Y}\left(G\left(x\right)\right)\right)\right], \end{array}$$

and

(12) $$\begin{array}{ccc} \ell_{\mathrm{GAN}}\left(H,\phi_{X}\right) & = & E_{x∼p_{X}\left(x\right)}\left[\log\phi_{X}\left(x\right)\right]\\ & & +E_{y∼p_{Y}\left(y\right)}\left[\log\left(1-\phi_{X}\left(H\left(y\right)\right)\right)\right]. \end{array}$$

As mentioned earlier, LS‐GAN [26] was employed as the adversarial loss for the stability of the network training. Moreover, $\ell_{\mathrm{cycle}}$ and $\ell_{\mathrm{identity}}$ are given by:

(13) $$\begin{array}{ccc} \ell_{\mathrm{cycle}}\left(G,H\right) & = & E_{x∼p_{X}\left(x\right)}\left[∥H\left(G\left(x\right)\right)-x{∥}_{1}\right]\\ & & +E_{y∼p_{Y}\left(y\right)}\left[∥G\left(H\left(y\right)\right)-y{∥}_{1}\right], \end{array}$$

and

(14) $$\begin{array}{ccc} \ell_{\mathrm{identity}}\left(G,H\right) & = & E_{y∼p_{Y}\left(y\right)}\left[∥G\left(y\right)-y{∥}_{1}\right]\\ & & +E_{x∼p_{X}\left(x\right)}\left[∥H\left(x\right)-x{∥}_{1}\right]. \end{array}$$

**FIGURE 7:**
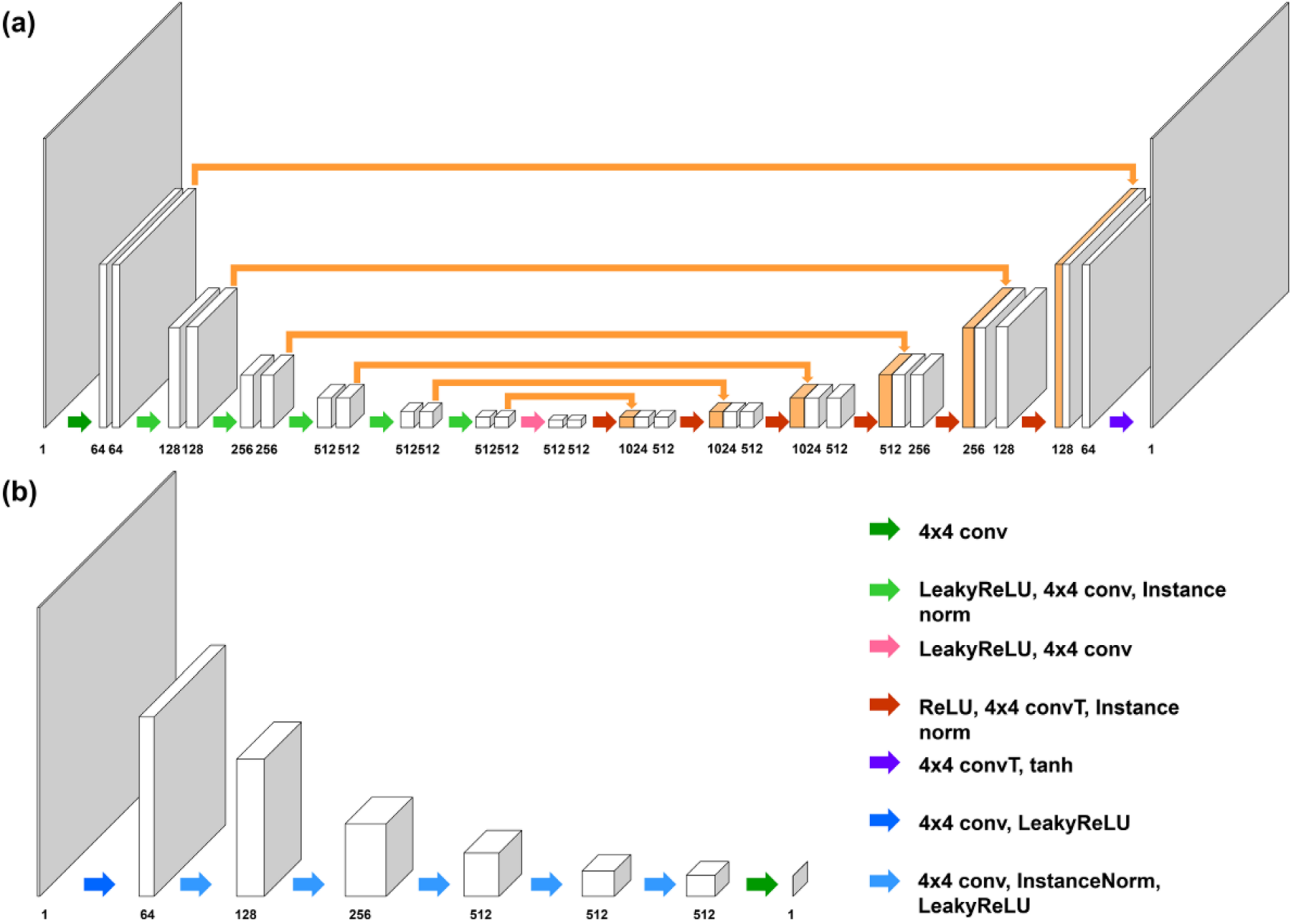
(a) Network architecture of *G* and *H*. (b) Network architecture of $\phi_{X}$ and $\pi_{Y}$.

We employ U‐net architecture for the generators G and H as shown in Figure 7a. The generator consists of six skip connection blocks, including 4 *×* 4 convolutional layer, instance normalization layer [32], and activation. We employ LeakyReLU and ReLU as activation functions for the encoders and the decoders, respectively. The number of convolutional filters was set to 64 at the early encoding stage and increased to 1,024 in the last stage by doubling the number of filters. The convolution kernel with stride two and the transposed convolution kernel with stride two were used for the pooling and unpooling operations. The final output of the network is provided after a tanh activation. For the discriminators $\phi_{X}$ and $\pi_{Y}$, we adopt 70 *×* 70 PatchGAN discriminator from [29] as shown in Figure 7b. The first layer contains 64 sets of 4 *×* 4 convolutional layers. Subsequently, the feature maps are processed by the five sets of the PatchGAN discriminator block, consisting of 4 *×* 4 convolution layer, instance normalization layer, and LeakyReLU activation. At each stage, the number of convolution kernels was doubled until it reached 512. In the last layer, a single 4 *×* 4 convolution layer was employed to provide the output.

Our model was trained with Adam optimizer [33] using a single batch. The model is trained for the first 100 epochs with the initial learning rate of 2 × 10^−5^. Over the next 400 epochs, the learning rate was linearly decreased to 0. The individual image was normalized to the range [0,1] for pre‐processing. We set the weights for the losses as follows: α=8,β=1, and γ=0.8. All the experiments were implemented with PyTorch library [34] on an NVIDIA GeForce RTX 3090 and it took about two days. The further detailed information for network training in provided in Section S8.

### 4.4 Statistical Analysis

To evaluate the statistical significance of performance differences in CNT junction detection, a Student's t‐test was conducted comparing three annotators: a researcher, a student annotator, and the proposed algorithm. The analysis was conducted on five independent samples shown in Figure 5, with two‐sided significance testing. As presented in Table 4, the proposed algorithm demonstrated statistically superior performance compared to both human annotators. When compared to researcher annotations, our algorithm showed significant improvements in sensitivity (*p <* 0.001) and IoU (*p* = 0.004). Similar superiority was consistently observed in comparison to student annotations across all metrics: precision (*p* = 0.016), sensitivity (*p* = 0.008), and IoU (*p* = 0.003). These results establish the statistical advantage of the deep learning‐based CNT extraction algorithm over manual annotation. Furthermore, the proposed method exhibited lower standard deviation in CNT junction detection across the five independent samples compared to human annotators, confirming its robustness across diverse samples.

**TABLE 4:** Annotator characteristics and statistical results of CNT junction detections. Data are presented mean ± standard deviation. Significant improvements (*p <* 0.05) is highlighted in bold.

	Annotator	Mean ± SD	P‐value (vs Proposed)
Precision	Researcher	57 % ± 15.3	**0.053**
	Student	70 % ± 10.9	0.016*
	Proposed	83 % ± 8.6	—
Sensitivity	Researcher	47 % ± 9.2	*<* 0.001*
	Student	52 % ± 9.8	0.008*
	Proposed	79 % ± 8.4	—
IoU	Researcher	33 % ± 7.1	0.004*
	Student	34 % ± 7.6	0.003*
	Proposed	57 % ± 5.2	—

## Conflicts of Interest

The authors declare no conflict of interest.

## Supporting information




**Supporting File**: advs73303‐sup‐0001‐SuppMat.pdf.

## Data Availability

The data that support the findings of this study are available from the corresponding author upon reasonable request.
