# Supplementary material for: Unpaired Learning‐Enabled Nanotube Identification from AFM Images
Source: Adv Sci (Weinh). 2025 Dec 25;13(12):e12504. doi: 10.1002/advs.202512504 (PMC12948285; doi:10.1002/advs.202512504)
Supplement: Supplementary file 1 — Supporting File: advs73303‐sup‐0001‐SuppMat.pdf. [file ADVS-13-e12504-s001.pdf]

Unpaired Learning for Robust Nanotube Identification from AFM Topography Images : Supporting Information

SOYOUNG NA[1,†], SOOBIN PARK[1,†], YOUNSU JUNG[2], JINHWA PARK[2], JIMIN HONG[1], JIHYUN LEE[1] ALBERT KIM[3], BONGJUN KIM[1], EUNJU CHA[1,*], GYOUJIN CHO[2,*], SEUNG HYUN SONG[1,*]

[1]*Department of Electrical Engineering, Sookmyung Women's University, Seoul, South Korea*
[2]*Department of Biophysics, Institute of Quantum Biophysics, Sungkyunkwan University, Suwon, South Korea*
[3]*Department of Medical Engineering, University of South Florida, Tampa, FL, U.S.A.*
[†]*These authors contributed equally.*
[*]*Corresponding authors: eunju.cha@sookmyung.ac.kr, gcho1004@skku.edu, shsong.ee@sookmyung.ac.kr*

S1. PREVIOUS CNT NETWORK MORPHOLOGY EXTRACTION METHOD

The image processing was conducted using the 'OpenCV' library in Python through successive steps. Firstly, the AFM topography image was low-pass-filtered in the frequency domain to eliminate the fast-scan line. Subsequently, the height data obtained from AFM is converted to an 8-bit format. The image was then converted to a binary representation using the local Otsu method to classify individual pixels as either CNT or dielectric. After average filtering, we identified the CNT junctions by applying a global threshold and further classified the junctions into aggregates and non-aggregates based on the size of the junctions. Lastly, the 'Contour' function was used to quantify the sizes and the positions of the junctions, allowing for the extraction of morphology parameters, including junction density, mean distance between junctions, mean CNT height, and degree of aggregation (Supplementary Figure S1(a)). Supplementary Figure S1(b)-(g) illustrate the representative intermediate images obtained during the feature extraction process. The binary image notably enhances the contrast between CNT and dielectric compared to the original AFM topography image (Supplementary Figure S1(b)-(c)). Subsequent images (Supplementary Figure S1(d)-(e)) demonstrate the emphasized junctions, while most slender wire-like features (i.e., individual CNTs) are eliminated. The identified junctions and aggregates are depicted in Supplementary Figure S1(f)-(g) by outlining their contours.

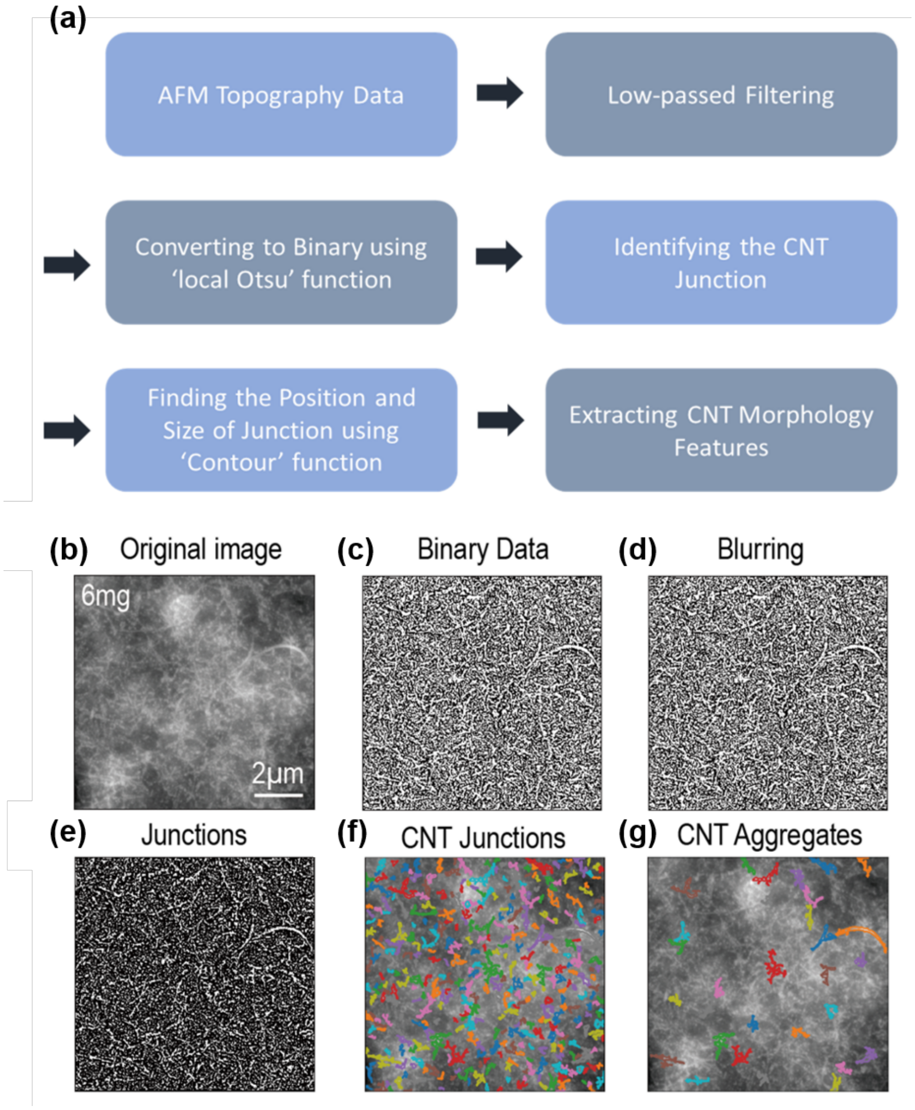

Fig. S1. (a) AFM topography data images passed low-passed filtering to eliminate noise, and converting binary using 'local Otsu' function. After average filtering, identifying the CNT junction by applying a global threshold. Finally, the 'Contour' function was used to quantify the size and position of the junctions. (b) Original AFM image of 6mg concentration. (c) The binary image distinguishing between the substrate and CNTs, with white represents CNTs and black signifies the substrate. (d) - (e) Images that after applying average filtering, CNT junctions were extracted using global threshold.(f) - (g) Visualizing the outlines of CNT junctions and aggregations in an image.

S2. AFM TOPOGRAPHY IMAGES OF CNT + SUBSTRATE AND SUBSTRATE

Characterization of the CNT network morphology was challenging, since the heterogeneity of the images also makes the training of 'CNT+Substrate' images very difficult as shown in Supplementary Figure S2(a)-(d). In contrast, Supplementary Figure S2(e)-(h) demonstrates that AFM images with only the dielectric substrates are relatively heterogeneous compared to the CNT images.

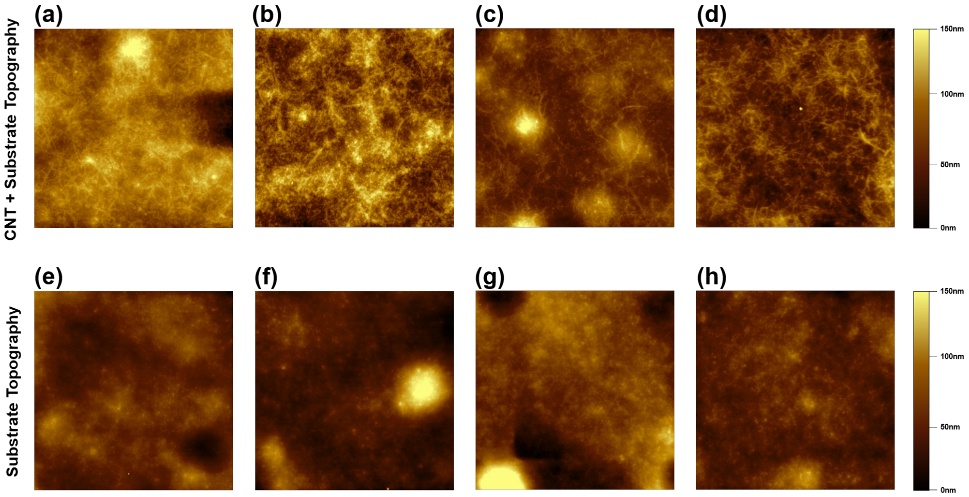

Fig. S2. (a)-(d) AFM topography images of the substrates with the CNT network morphology can be highly heterogeneous depending on the presences of CNT aggregates and contaminants. In the contrast, (e)-(h) AFM topography images of the pure dielectric substrates are relatively homogeneous.

S3. CNT NETWORK MORPHOLOGY AND ELECTRICAL PERFORMANCE

The representative AFM images of variable CNT concentrations are illustrated in the Supplementary Figure S3(a), and show clear qualitative differences in the morphology of the CNT network depending on the CNT concentration. In addition, the I-V curve also varies with the CNT concentration. As the concentration of CNT in the ink increases, both the on-current and off-current increase, while the on / off ratio decreases (Supplementary Figure S3(b))

(a)

(b)

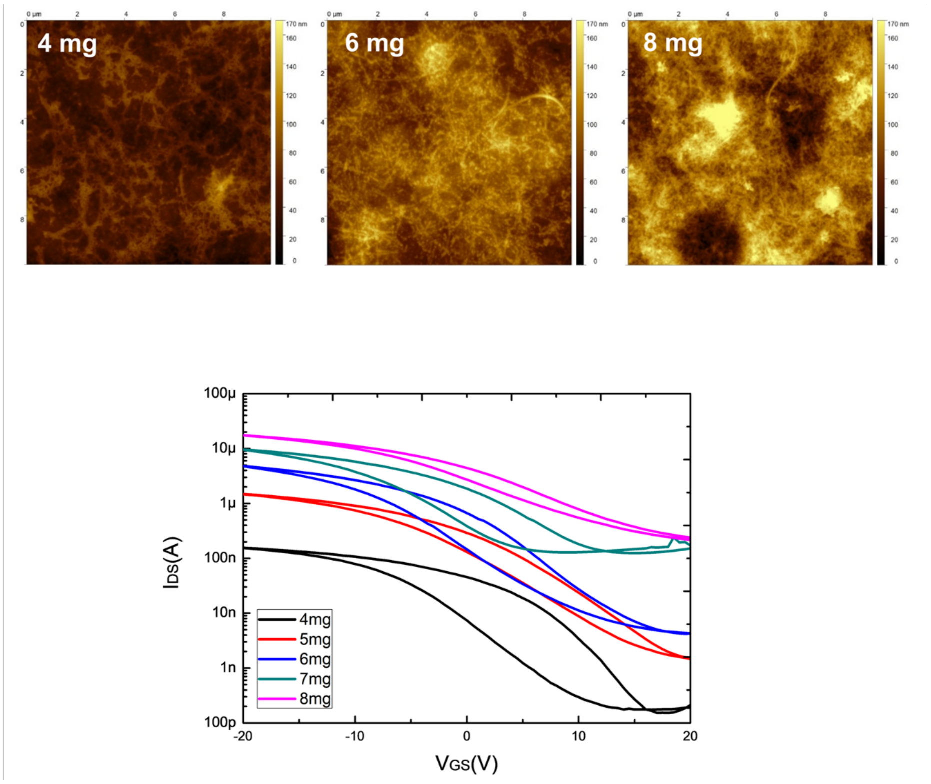

Fig. S3. CNT AFM images corresponding to varying CNT concentration and associated I-V curves (a) Qualitative differences in CNT network morphologies with increasing CNT density. (b) And corresponding changes in I-V curves, reflecting an increases on/off currents and a degradation on-off ratio.

S4. INVESTIGATING COMPETING INTERACTIONS IN CNT NETWORK FORMATION

To investigate the experimental results of the effects of substrate surface energy on the CNT network morphology, we prepared Si wafers subjected to three distinct treatments aimed at altering surface energy. Surface energy manipulation was achieved on an SI wafer through the application of APTES, plasma treatment, and an untreated reference. Following this, CNTs were inkjet-printed in layers ranging from 1 to 5. Notably, distinct CNT dispersion, alignment, and aggregation behaviors were observed in the AFM image of the single-layer deposition, showcasing variations corresponding to the surface treatment(Supplementary Figure S4). With an increase in the number of printed layers, a phenomenon resembling CNT clustering emerged(Supplementary Figure S5).

With this insight, we carried out ab initio calculations by maintaining the CNT-CNT interaction fixed ($W_{CNT-CNT} = 1$) and adjusting the Sub-CNT interaction ($W_{Sub-CNT}$). Specifically, $W_{Sub-CNT}$ was set to 0, 1, 10, 20, and 50, respectively, and the CNT network was examined for each corresponding energy level. Upon depositing CNTs for the first time, their alignment tends to reduce the total energy, resulting in a decrease in $L_{Sub-CNT}$ as $W_{Sub-CNT}$ increases. Consequently, this prompts CNT aggregation. Additionally, observations from simulations with varying quantities of CNTs reveal that an increase in quantity doesn't encourage CNT alignment in vacant spaces; instead, the CNTs tend to aggregate more among themselves (Supplementary Figure S6), consistent with the experimental results.

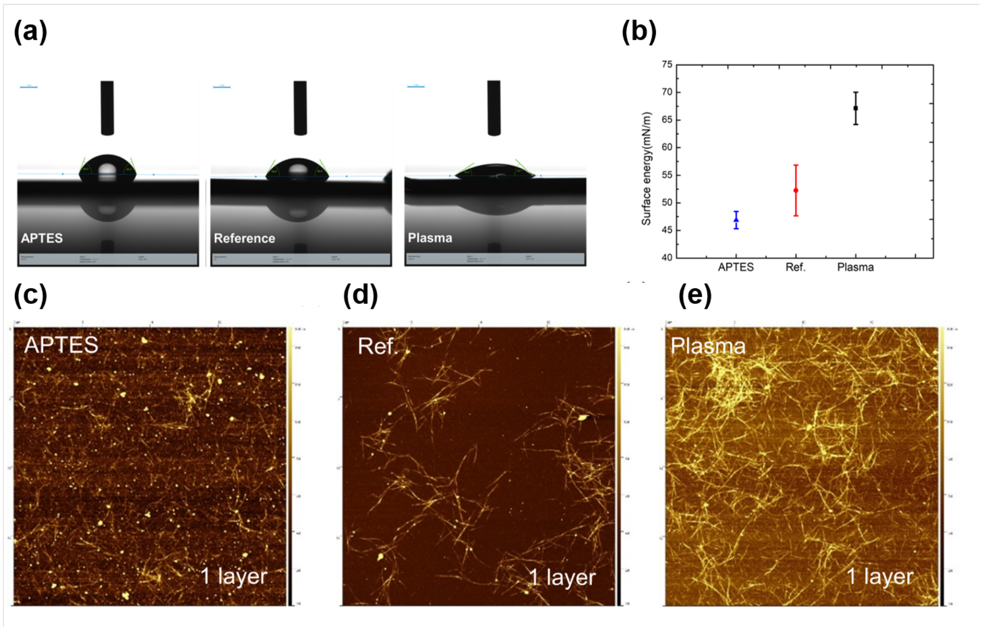

Fig. S4. Changes in CNT network morphology based on Surface energy. (a) - (b) Various treatments (APTES, Plasma and Untreated) on the Si substrate enable the modulation of surface energy. (c) – (e) Differences in CNT network morphology based on surface energy can be observed through AFM images

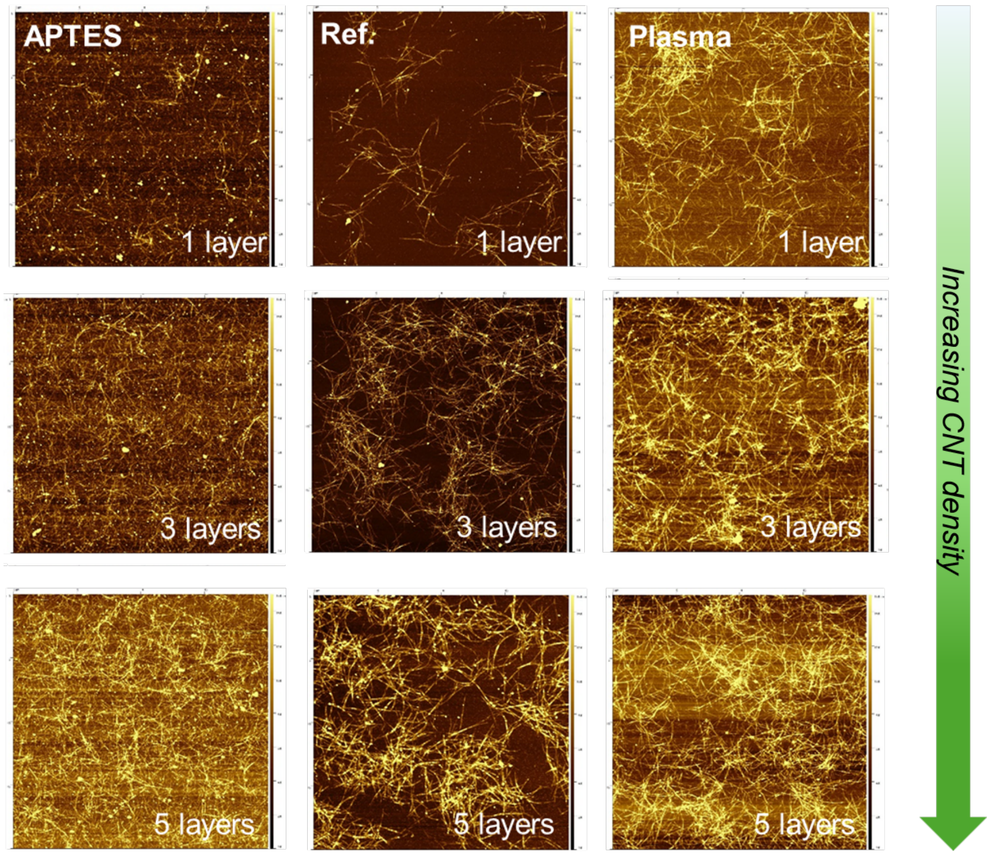

Fig. S5. The various CNT network morphology (APTES, Plasma and untreated) AFM images by repeated printing. The morphology of CNT deposition varies based on the treatment, particularly showcasing increased agglomeration as printing procedures continue.

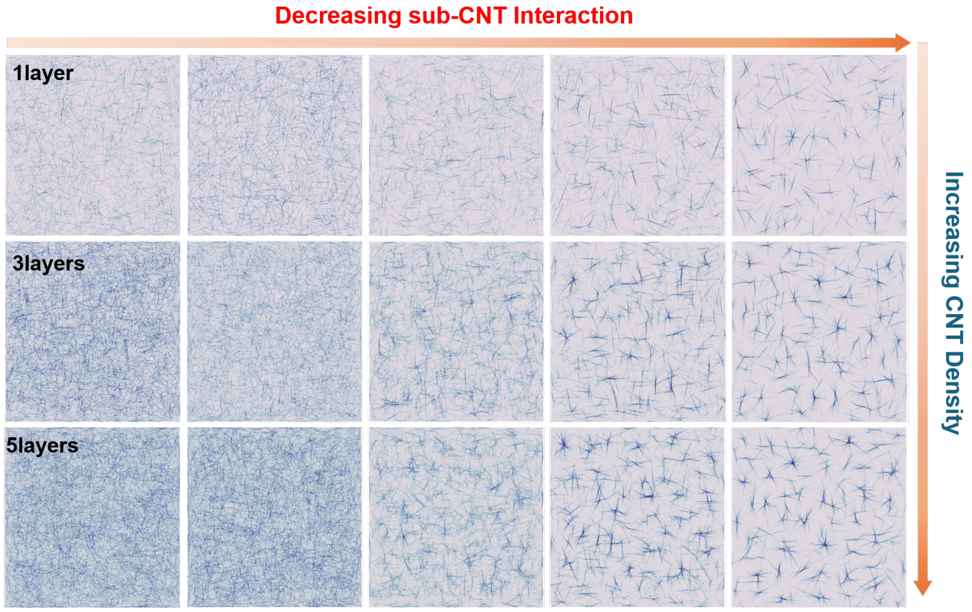

Fig. S6. Ab initio Study of CNT Network Morphology with WSub-CNT / WCNT-CNT Interactions. As Sub-CNT interactions decrease, enhanced aggregation of CNTs can be observed. And above a certain threshold of CNT densities, the aggregation becomes increasingly prominent.

S5. COMPARISON BETWEEN ACTUAL CNT AFM TOPOGRAPHY IMAGE AND SIMULATED CNT IMAGE

The ground-truth images were generated by adjusting the length, quantity and energy of CNTs via ab initio calculations. By combining these with the actual substrate AFM images, simulated CNT images are generated that are closely similar to the actual AFM images of CNTs.

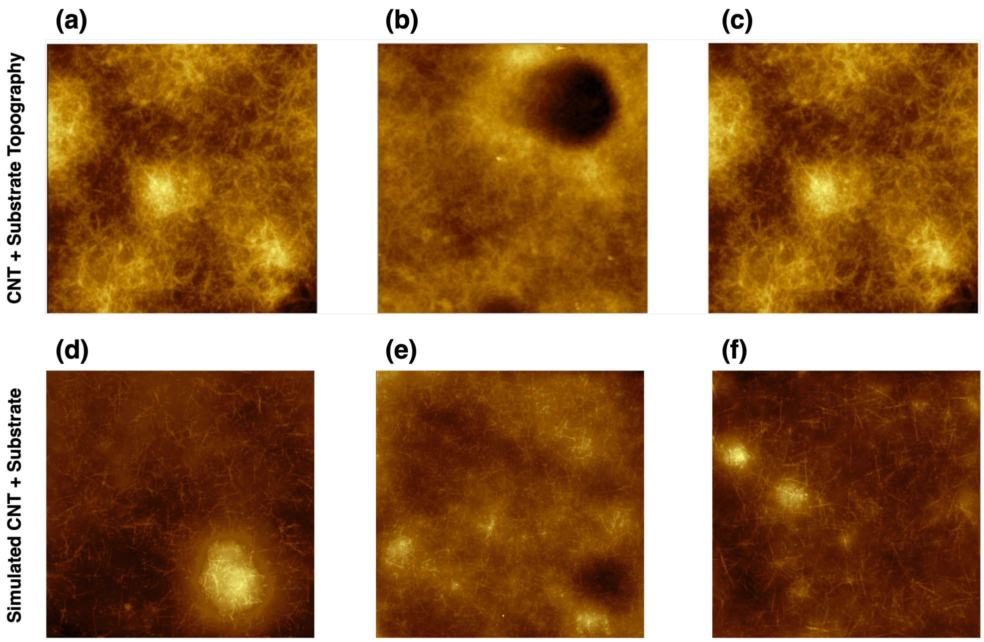

Fig. S7. (a)-(c) The AFM topography images of CNT network morphology. (d)-(f) The ground truth CNT network images

S6. COMPARISON METHODS

As the comparative methods, the conventional Otsu-based method [1], cycleGAN [2], and the supervised learning-based method were employed.

S6.1 Otsu-based Method

The Otsu-based AFM image processing [1] was performed according to the methodology described in SI. Previous CNT network morphology extraction method. The optimal parameters for the processing were determined through the manual investigation. Specifically, the radius of local Otsu mask and the threshold for binarization were set to 7 pixels and 1.05, respectively. The resulting binary mask was multiplied by the corresponding measurements to capture the CNT networks.

S6.2 CycleGAN

To validate the role of the proposed loss function ℓ_{pos}, the original cycleGAN [2] model was employed as the comparison study. For a fair comparison, we adopted the same network architectures for the original cycleGAN. The objective function to train the cycleGAN model is as follows:

$$\ell(G, H, \phi_{\mathcal{X}}, \pi_{\mathcal{Y}}) = \ell_{GAN}(G, \pi_{\mathcal{Y}}) + \ell_{GAN}(H, \phi_{\mathcal{X}}) + \alpha \ell_{cycle}(G, H) + \beta \ell_{identity}(G, H), \tag{S1}$$

where α and β are hyper-parameters. Note that ℓ_{pos} in Eq. (2) was not utilized when training the cycleGAN model, while the rest of the loss functions such as ℓ_{GAN}, ℓ_{cycle}, and $\ell_{identity}$ were same as defined in Eq. (11), (12), (13) and (14). The hyper-parameters α and β were set to 8 and 1, which were identical settings for training the proposed model. The flowchart for the original cycleGAN is illustrated in Figure S8.

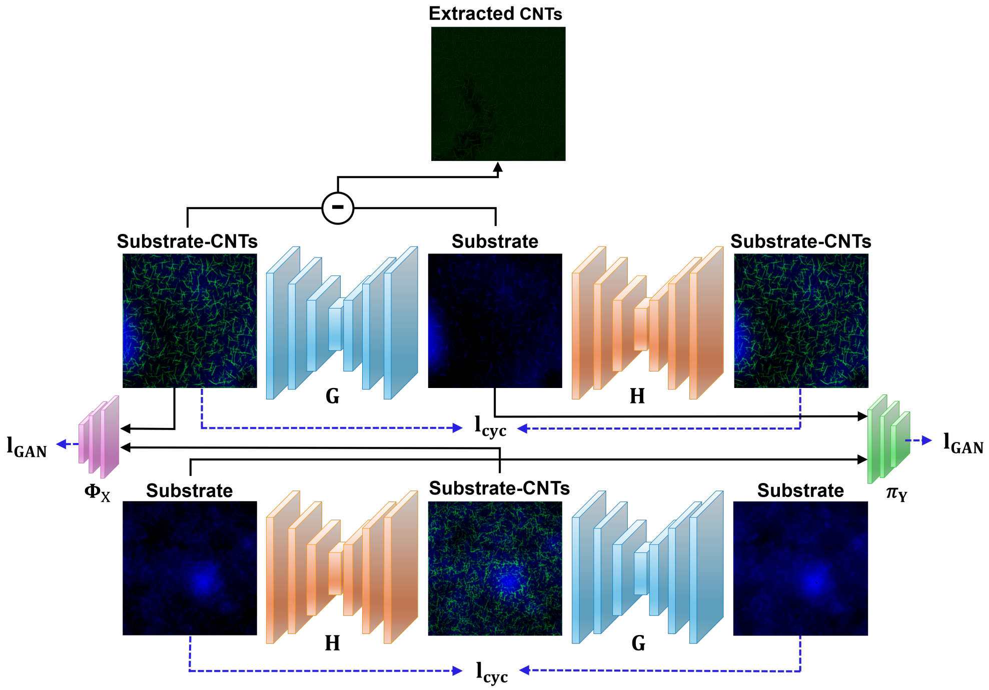

Fig. S8. Flowchart of the cycleGAN [2] model to extract CNT networks

S6.3 Supervised learning

The simulation experiments enabled the creation of a paired dataset consisting of images of substrates with CNTs, $\{x_i\}_{i=1}^N$, and the corresponding images of CNT networks, $\{z_i\}_{i=1}^N$. Therefore, the network can be trained based on supervised learning using the paired dataset. Specifically, the network F learns the direct mapping from the substrate with CNTs to CNT networks by solving the optimization problem as follows:

$$\min_F \ell(F(x_i), z_i), \tag{S2}$$

where

$$\ell(F(x_i), z_i) = \frac{1}{N} \sum_{i=1}^{N} \|F(x_i) - z_i\|_2^2. \tag{S3}$$

For a fair comparison, the same network architecture of G and H was used for F, and hyper-parameters such as learning rate and optimizer were also employed with the same settings. It is worth noting that supervised learning requires a paired dataset, which is challenging to acquire in many scenarios.

S7. PERFORMANCE COMPARISON OF OUR MODEL WITH HUMAN OPERATORS ON SIMULATED IMAGES

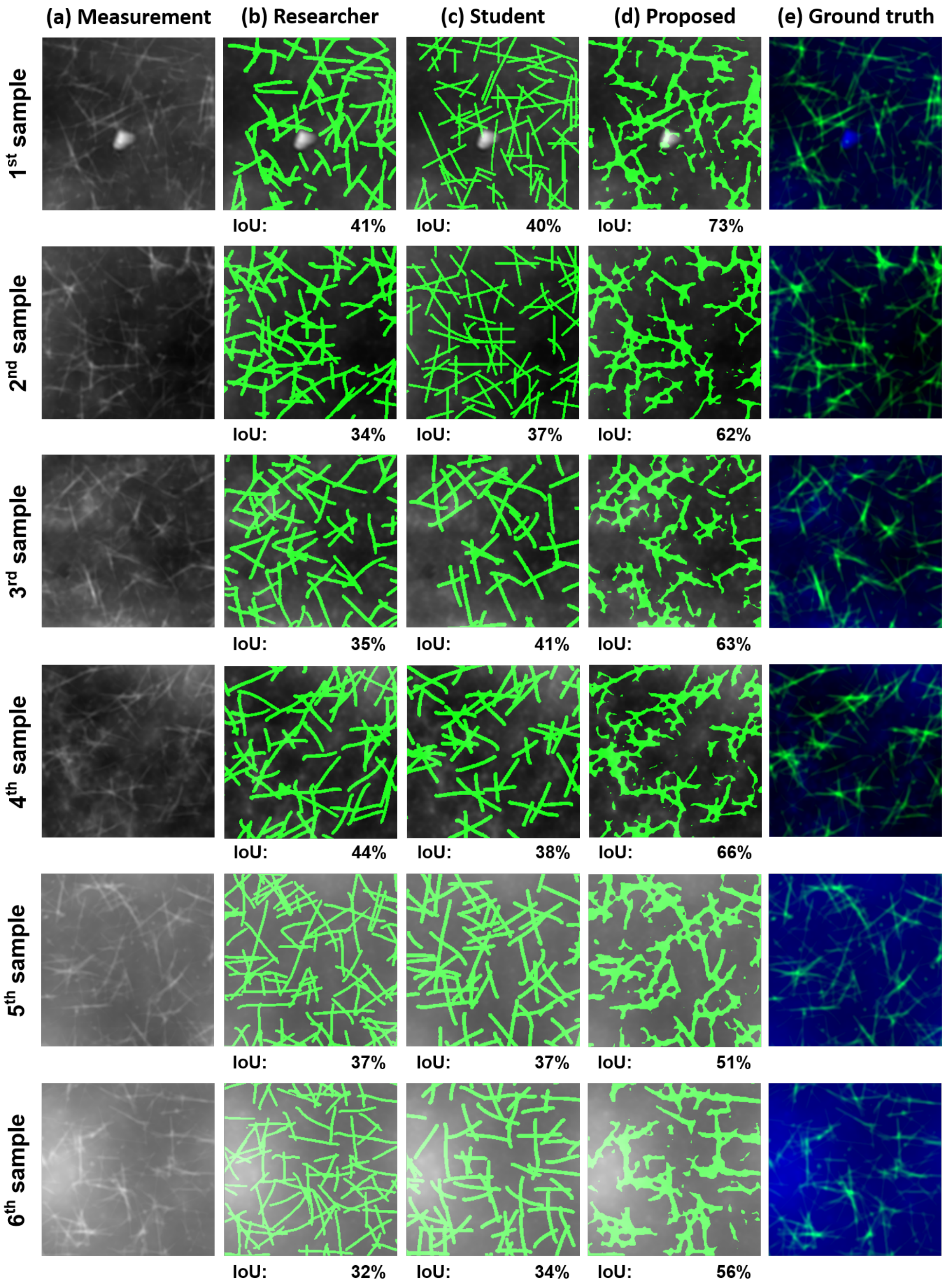

Fig. S9. Performance comparison of CNT extraction from simulated images. (a) Measurements. (b) Results from an expert researcher. (c) Results from a student operator. (d) Results from our proposed algorithm. Manual detection consistently shows inferior performance compared to the proposed method in identifying CNT junctions on rough and flexible substrates.

Figure S9 presents the CNT extraction results from simulation data, comparing the performance of our model with that of human operators. For a quantitative evaluation, we calculated the Intersection over Union (IoU) in a pixel-wise manner. As shown in Figure S9(d), our model achieves extraction results that are nearly identical to the ground-truth in Figure S9(e). In

particular, it is worth noting that the proposed method demonstrates consistent performance across samples with varying levels of roughness. The roughness of the substrate, which has been a critical challenge for human operators and traditional image processing methods in AFM data analysis, is effectively addressed by our model. Moreover, unlike human operators, the model is able to estimate the width of CNTs, which increases the precision of the predictions. This capability contributes to the observed high IoU scores.

S7.1 Analysis of model performance on simulation data

Figure S10(a) presents box plots of accuracy, sensitivity, specificity, and precision for both the proposed model and a supervised learning-based CNT analysis model. Numerous outliers are observed, which can be attributed to substrate features being misclassified as CNTs due to surface contamination. To investigate this, we compare two substrate images from the same scan in Figure S10(b): a contaminated sample (1st sample) and a relatively clean one (2nd sample). AFM phase analysis reveals a broader and double-peaked distribution in the 1st sample, while the 2nd sample exhibits a narrow Gaussian distribution. These findings imply that surface contaminants contribute significantly to performance degradation.

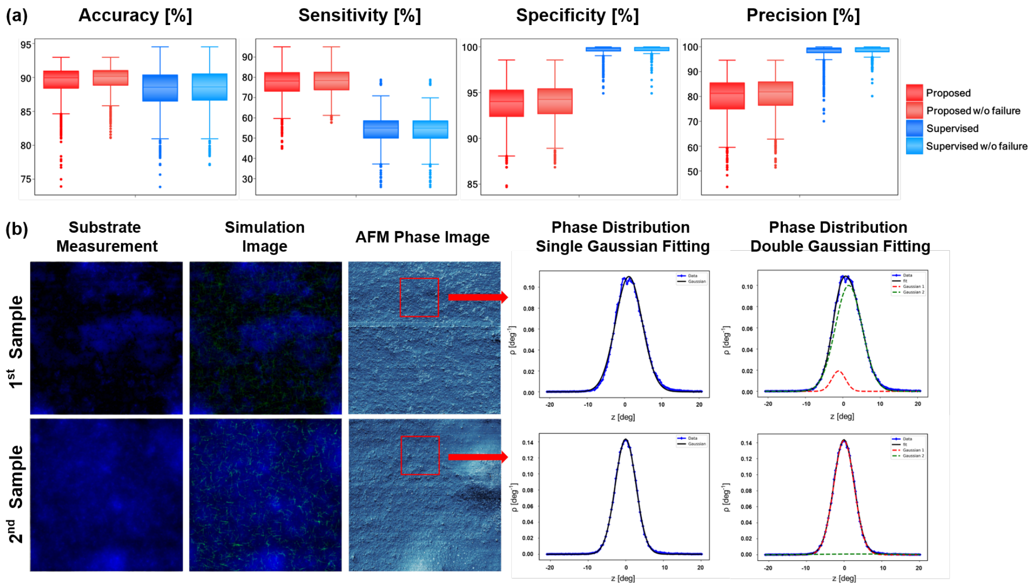

Fig. S10. (a) Quantitative comparison using simulation dataset. (b) Comparison of AFM phase distributions at identical substrate positions: the 1st sample showing good model performance exhibits a double-peak structure, while the 2nd sample showing degraded model performance is fitted by a single Gaussian, indicating reduced phase contrast.

S7.2 Ablation study on the threshold value to generate the mask M_{cnt}

For fair quantitative evaluation, we conducted ablation study using simulation data with ground-truth binary masks M_{cnt}. To generate binary masks from the model predictions, thresholding is applied to the continuous CNT images. As shown in Figure S11, depending on the threshold for generating the binary mask, there is a trade-off between sensitivity and specificity: a higher threshold retains only high-confidence pixels as the CNT regions, reducing false positives but potentially increasing false negatives, whereas a lower threshold captures more subtle features at the cost of higher false positive rates. This allows users to adjust the detection strategy according to their specific analysis requirements. We chose a threshold of 0.15 to achieve high specificity, minimize false positive network extraction, and maintain sufficient sensitivity to accurately detect existing CNTs.

S8. DETAILED PROTOCOL

Table S1 summarizes the training configurations for our framework. It provides details such as dataset size, network architectures, training hyperparameters, and inference speed.

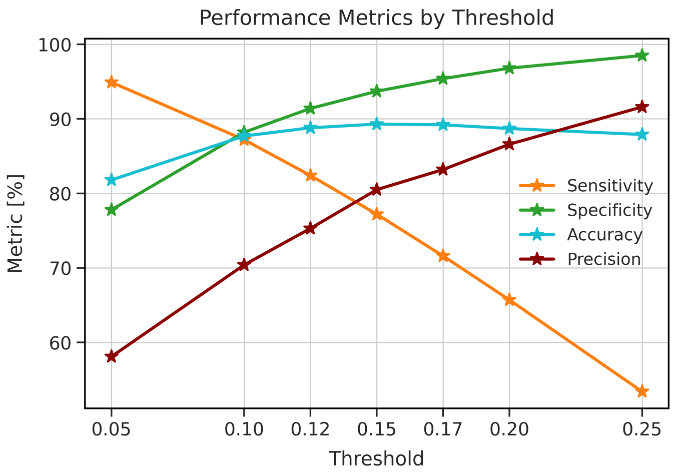

Fig. S11. Performance metrics on simulation data for varying threshold values. The ablation study demonstrates the trade-off between sensitivity and specificity.

Elements	Description
**Dataset Construction**	
Number of Substrate-CNTs dataset (domain $\mathcal{X}$)	3300
Number of Substrate dataset (domain $\mathcal{Y}$)	3300 (augmented from 33 images)
**Training**	
Generator (G) Network Architecture	U-Net
Discriminator (D) Network Architecture	70×70 PatchGAN Discriminator [3]
Optimizer	Adam Optimizer [4]
Number of Training Epochs	500
Learning Rate Scheduling	2×10^{-5} for 100 epochs then linearly decreased to 0 for the rest.
Hyper-parameters	$\alpha = 8, \quad \beta = 1, \quad \gamma = 0.8$
**Inference**	
Extraction of CNT network morphology	$x_{cnt} = x - G^*(x).$
Inference Speed	0.4 s per 256×256 sample

Table S1. Detailed configurations for dataset construction, training, and inference.

REFERENCES

1. B. Mirka, N. A. Rice, P. Williams, *et al.*, "Excess polymer in single-walled carbon nanotube thin-film transistors: its removal prior to fabrication is unnecessary," ACS nano **15**, 8252–8266 (2021).
2. J.-Y. Zhu, T. Park, P. Isola, and A. A. Efros, "Unpaired image-to-image translation using cycle-consistent adversarial networks," in *Proceedings of the IEEE international conference on computer vision*, (2017), pp. 2223–2232.
3. P. Isola, J.-Y. Zhu, T. Zhou, and A. A. Efros, "Image-to-image translation with conditional adversarial networks," in *Proceedings of the IEEE conference on computer vision and pattern recognition*, (2017), pp. 1125–1134.
4. D. P. Kingma and J. Ba, "Adam: A method for stochastic optimization," arXiv preprint arXiv:1412.6980 (2014).
